# Progress in copper-catalyzed trifluoromethylation

**DOI:** 10.3762/bjoc.14.11

**Published:** 2018-01-17

**Authors:** Guan-bao Li, Chao Zhang, Chun Song, Yu-dao Ma

**Affiliations:** 1 School of Pharmaceutical sciences, Shandong University, 44 West Culture Road, Jinan 250012, PR China; 2 Department of chemistry, Shandong University, 27 Shanda South Road, Jinan 250100, PR China

**Keywords:** copper, fluorine, trifluoromethylation

## Abstract

The introduction of trifluoromethyl groups into organic molecules has attracted great attention in the past five years. In this review, we describe the recent efforts in the development of trifluoromethylation via copper catalysis using nucleophilic, electrophilic or radical trifluoromethylation reagents.

## Introduction

**Figure 1:**
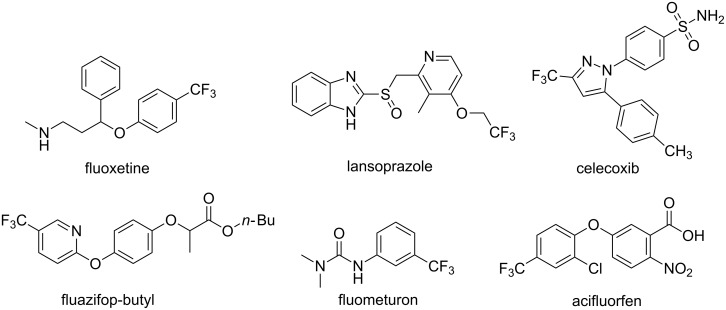
Selected examples of pharmaceutical and agrochemical compounds containing the trifluoromethyl group.

The fluorine atom has a strong electronegativity and a small atomic radius, and the incorporation of fluoroalkyl groups into molecules imparts a variety of features. The trifluoromethyl group, as the most significant common used fluoroalkyl group, could improve molecular properties such as metabolic stability, lipophilicity and permeability [1–4]. Therefore, organic molecules bearing trifluoromethyl groups are widely used in pharmaceuticals and agrochemicals, such as the antidepressant fluoxetine, the anti-ulcer drug lansoprazole and so on (Figure 1).

The specific roles of the trifluoromethyl group (CF_3_) in biologically active molecules promote the development of novel methods to construct C–CF_3_ bonds in the past few years. Among the many methods developed, copper-catalyzed trifluoromethylation has gained enormous interest due to its high efficiency and cheapness.

Recently, several reviews on trifluoromethylation were disclosed. Xu, Dai [5] and Shen [6] mainly discussed progress in copper-mediated trifluoromethylation before 2013. Other works focus on the trifluoromethylation of alkynes [7] or on the C(sp^3^)−CF_3_, C(sp^2^)−CF_3_, and C(sp)−CF_3_ bond construction [8]. This minireview mainly focuses on the copper-mediated or -catalyzed trifluoromethylations from 2012 to 2016. And some previous pioneering works were included to gain a comprehensive understanding of the development of the diverse synthetic methods over the time. Throughout this minireview, compounds **1** are the trifluoromethylation reagents.

## Review

### Copper-catalyzed trifluoromethylation of aryl and alkyl halides

The first example of copper-promoted perfluoroalkylation of aromatic halides was presented in a US patent 1968 [9]. Since then, the copper-catalyzed trifluoromethylation of aromatic compounds has entered a stage of rapid development. Many reviews [6,10] have been published on this subject, so this part mainly discussed trifluoromethylations using several new trifluoromethylation reagents and some important examples using traditional trifluoromethylation reagents were also involved.

#### Trifluoromethylation of aryl halides with traditional trifluoromethylation reagents and a trifluoromethyl-substituted sulfonium ylide as a new reagent

**Scheme 1:**
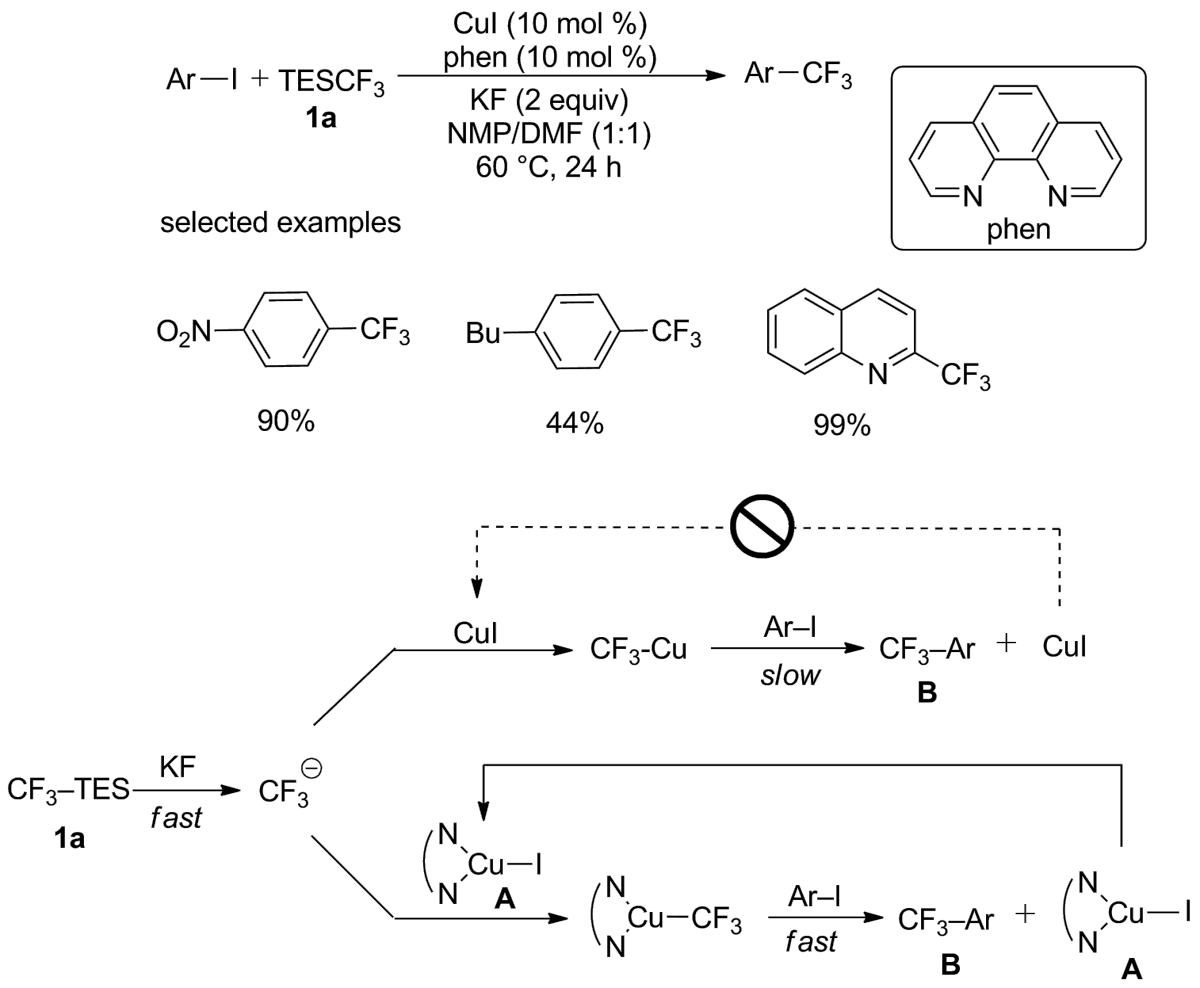
Introduction of a diamine into copper-catalyzed trifluoromethylation of aryl iodides.

The CuI-mediated cross-coupling protocol using TESCF_3_ was firstly reported by Urata and Fuchikami [11]. The proposed mechanism of this reaction was demonstrated in Scheme 1. But it was found that the generation rate of trifluoromethyl anions is much higher than the second step affording **B** and CuI. Thus, there is no sufficient recirculated CuI to react with trifluoromethyl before its decomposition. Optimizations were then performed to overcome this shortcoming in 2009 [12], a series of diamine ligands such as 1,10-phenanthroline (phen) were discovered. These diamines were able to accelerate the second step to regenerate sufficient amounts of reusable complexes **A** utilizing copper(I)-diamine complexes (Scheme 1), thus accelerating the trifluoromethylation of aryl/heteroaryl iodides.

Although this catalytic reaction worked efficiently, the trifluoromethyl source TESCF_3_ was expensive and relatively inaccessible, which made this process less economic, especially for large-scale synthesis.

**Scheme 2:**
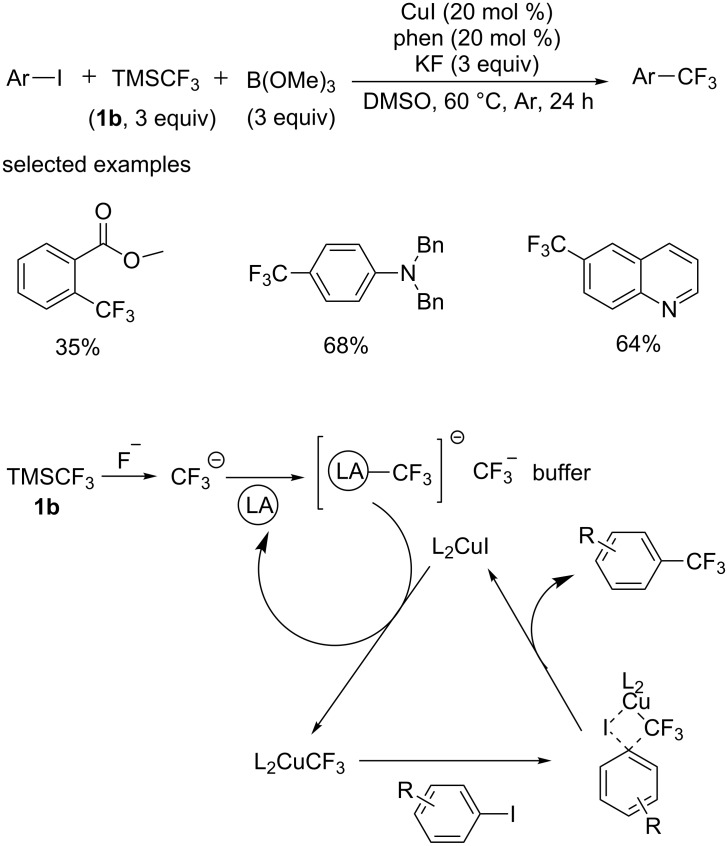
Addition of a Lewis acid into copper-catalyzed trifluoromethylation of aryl iodides and the proposed mechanism.

In 2014, Novák, Kotschy and co-workers [13] developed a new procedure with the relatively cheap and readily available TMSCF_3_ (Scheme 2). Similarly, the recycling of CuI was inefficient and led to the degradation of excessive trifluoromethyl anions. Differently, a novel strategy was developed to solve this problem, a Lewis acid such as trialkyl borate was added for the temporary trapping of the in situ generated trifluoromethyl anion and suppress its rapid decomposition (Scheme 2).

**Scheme 3:**
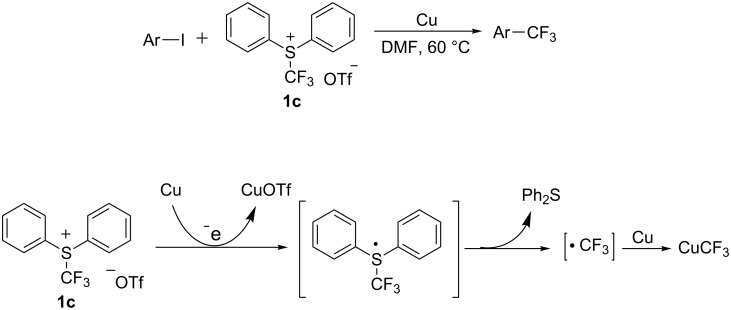
Trifluoromethylation of heteroaromatic compounds using *S*-(trifluoromethyl)diphenylsulfonium salts as a trifluoromethyl source.

*S*-(Trifluoromethyl)diphenylsulfonium salts, as commonly used electrophilic trifluoromethylation reagents, were orginally developed by the group of Yagupolskii [14]. When iodo-substituted aromatics and heteroaromatics were employed as the substrates, Xiao and co-workers [15] firstly used *S*-(trifluoromethyl)diphenylsulfonium salts in the presence of copper powder to convert the substrates into the corresponding trifluoromethylated compounds in high yield. The CuCF_3_ intermediate was formed in this process, as confirmed by ^19^F NMR spectroscopy and ESIMS. It was proposed that [CuCF_3_] was generated through reduction of *S*-(trifluoromethyl)diphenylsulfonium triflate by Cu^0^ through a single-electron transfer (SET) process (Scheme 3).

**Scheme 4:**
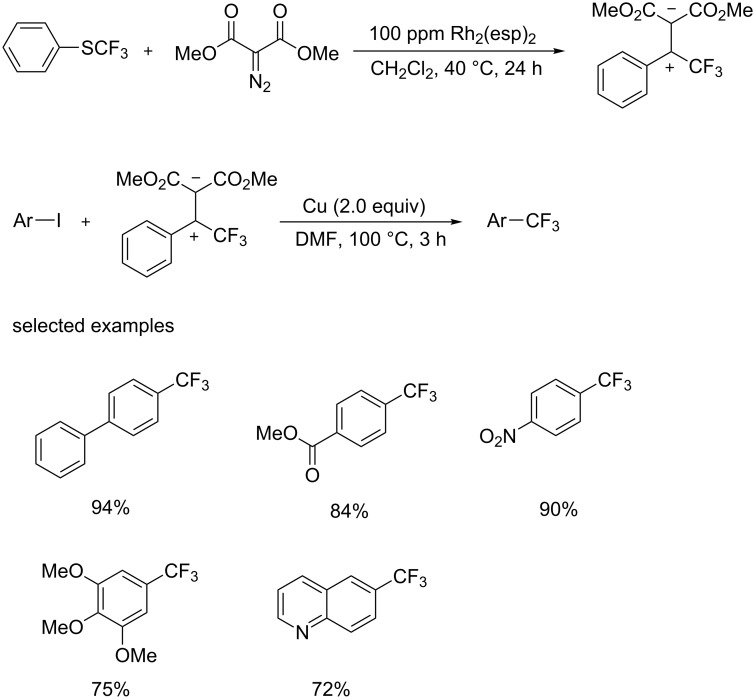
The preparation of a new trifluoromethylation reagent and its application in trifluoromethylation of aryl iodides.

In 2015, the group of Lu and Shen [16] developed a new electrophilic trifluoromethylation reagent, trifluoromethyl-substituted sulfonium ylide, which was prepared by a Rh-catalyzed carbenoid addition to trifluoromethyl thioether (Scheme 4). This process was conducted in dichloromethane at 40 °C for 4 h with a catalyst loading of 100 ppm. Moreover, this new reagent was easily scaled-up and stable to moisture and air. This reagent was applied in trifluoromethylation of aryl iodides. A variety of aryl and heteroaryl iodides were converted to the corresponding analogues in high yields. Both electron-donating and -withdrawing groups including methoxy, nitro and ester groups, were tolerated.

#### Trifluoromethylation of aryl halides using trifluoroacetates as the trifluoromethyl source

Reactions employing expensive electrophilic CF_3_ species such as Umemoto’s reagent or Togni’s reagent, were unpractical and limited on a large-scale synthesis. After comparison of the prices of different CF_3_ reagents, attention was paid on trifluoroacetates. Trifluoroacetate is readily available and one of the cheapest and most convenient sources of the trifluoromethylation for both industrial and medicinal purposes.

**Scheme 5:**
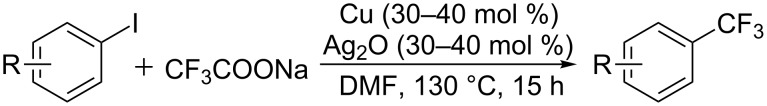
Trifluoromethylation of aryl iodides using CF_3_CO_2_Na as a trifluoromethyl source.

In 2011, a practical and ligand-free Cu-catalyzed decarboxylative trifluoromethylation of aryl iodides was reported by the group of Li and Duan, with sodium trifluoroacetate as the trifluoromethyl source and using Ag_2_O as a promoter (Scheme 5) [17].

**Scheme 6:**
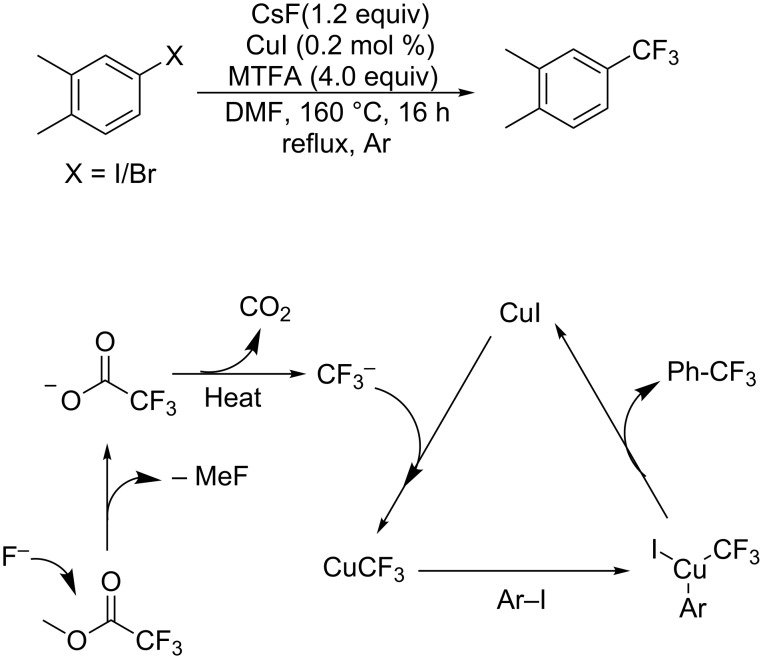
Trifluoromethylation of aryl iodides using MTFA as a trifluoromethyl source.

Subsequently, Beller and co-workers [18] finished a copper-catalyzed trifluoromethylation of aryl iodides with inexpensive methyl trifluoroacetate (MTFA) (Scheme 6). However, it was found that the generation of the trifluoromethyl anion proceeds faster than the subsequent transfer of CF_3_ to the aromatic halide. Subsequently, the problem was solved by using a slow addition mode that adjusted the rate of decarboxylation step to the rate of the consumption of CF_3_ in the aromatic trifluoromethylation step.

The attractive prospect of trifluoroacetate as the trifluoromethyl source for the preparation of trifluoromethylarenes prompted the investigation of the mechanism of this reaction. A mechanistic study indicated that CuCF_3_ was formed by decarboxylation of the trifluoroacetate (Scheme 6), followed by oxidative addition with aryl iodides and the trifluoromethylated products were delivered through reductive elimination.

**Scheme 7:**
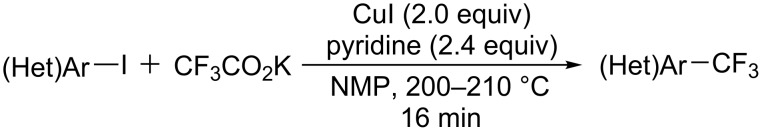
Trifluoromethylation of aryl iodides using CF_3_CO_2_K as a trifluoromethyl source.

Later, Buchwald and co-worker [19] firstly developed an efficient, scalable technique to introduce a trifluoromethyl group into aryl iodides with CF_3_CO_2_K as the CF_3_ source (Scheme 7). High temperature was required to accelerate the rate of the decarboxylation of CF_3_CO_2_K and the increased pressure occurred during the process, which brought problems under batch conditions. Buchwald and co-worker deal with it through combining flow chemistry. Under flow conditions, reaction parameters such as temperature, pressure and residence times could be readily managed. Besides, very short reaction times (16 minutes) were required to achieve full conversion of (hetero)aryl starting materials in this protocol.

**Scheme 8:**
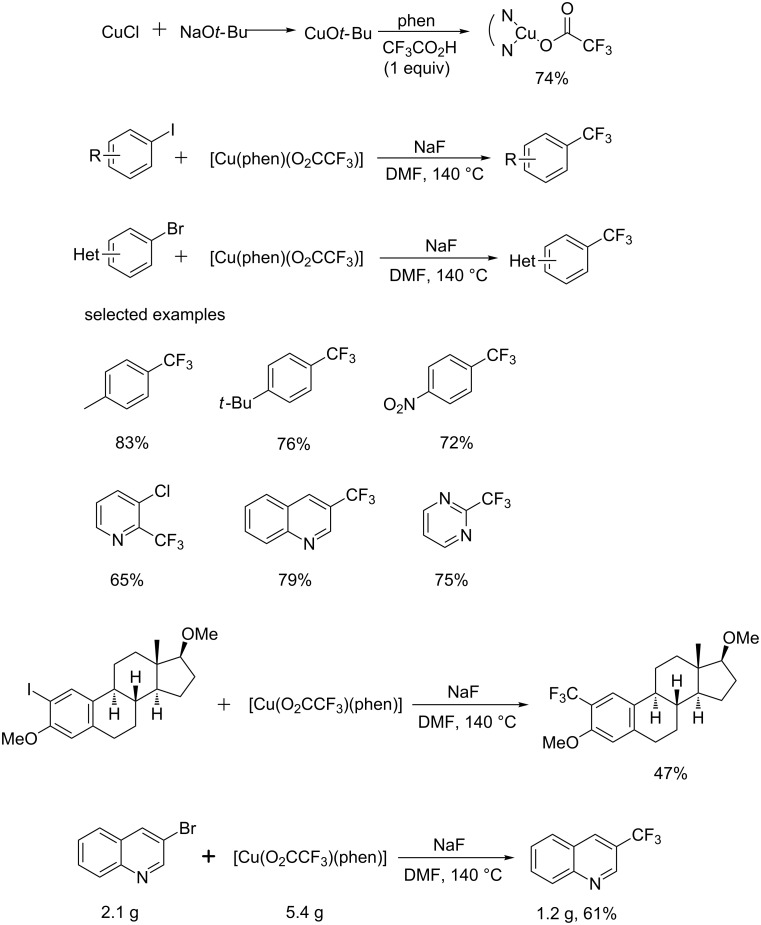
Trifluoromethylation of aryl iodides and heteroaryl bromides using [Cu(phen)(O_2_CCF_3_)] as a trifluoromethyl source.

More recently, Weng and co-workers [20] reported a new economic decarboxylative trifluoromethylation reagent [Cu(phen)O_2_CCF_3_], which was prepared from readily available and inexpensive starting materials (Scheme 8). Treatment of copper *tert*-butoxide with phen, followed by addition of trifluoroacetic acid afforded the air-stable [Cu(phen)O_2_CCF_3_] complex, which was characterized by X-ray crystallography. Aryl iodides, which contained nitrile, ketone, aldehyde, ester, methyl, phenyl groups, etc., were successfully reacted with this trifluoromethylation reagent to give the corresponding products in moderate to high yields. Also, a broad spectrum of heteroaryl bromides proceeded smoothly to form the corresponding products. A late-stage trifluoromethylation of an estradiol derivative and a gram-scale reaction were performed with this protocol demonstrating the applicability of this protocol to other pharmaceutically relevant molecules and its scalability.

#### Trifluoromethylation of aryl halides using difluorocarbene precursors as the trifluoromethyl source

The trifluoromethyl sources employed in the above-mentioned trifluoromethylations were mainly CF_3_-containing reagents. Besides, some examples employing difluorocarbene precursors as the trifluoromethylation reagents were reported recently.

**Scheme 9:**
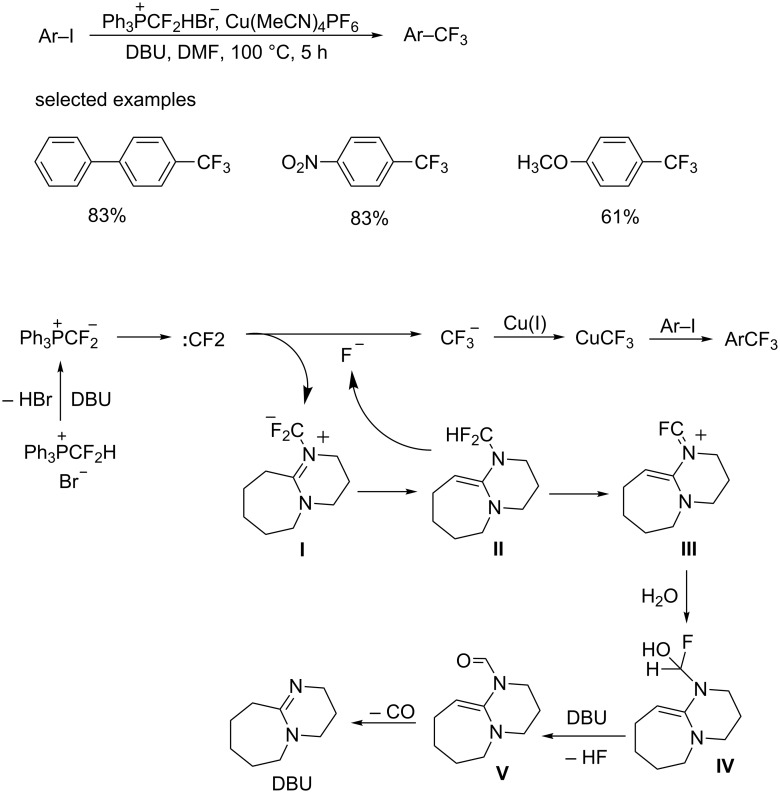
Trifluoromethylation of aryl iodides with DFPB and the proposed mechanism.

In 2015, the group of Lin, Zheng and Xiao [21] disclosed a trifluoromethylation reagent, the difluorocarbene precursor difluoromethyltriphenylphosphonium bromide (DFPB), which could be applied in the trifluoromethylation of aromatic iodides without addition of external fluoride (Scheme 9). It was found that DBU can promote the decomposition of difluorocarbene to give fluoride which then reacts with difluorocarbene to a trifluoromethyl anion. Both electron-rich and electron-deficient substrates were converted to the corresponding analogues in moderate to good yields, without producing byproducts from a pentafluoroethylation.

The proposed reaction mechanism is depicted in Scheme 9. First, a phosphonium ylide is formed after treating DFPB with DBU, and then dissociated to generate a difluorocarbene. The difluorocarbene reacts with DBU affording nitrogen ylide **I**, followed by a rearrangement to give unstable difluoromethyl amine **II**. Decomposition of **II** produces intermediate **III** by releasing a fluoride ion which was trapped by difluorocarbene affording the trifluoromethyl anion. The latter reacts with copper to CuCF_3_. The desired Ar–CF_3_ was formed by the reaction of CuCF_3_ with the aromatic iodide. Additionally, the intermediate **III** is also unstable and decomposes to intermediate **IV** in the presence of water. And DBU is regenerated after the elimination of HF and decarbonylation.

**Scheme 10:**
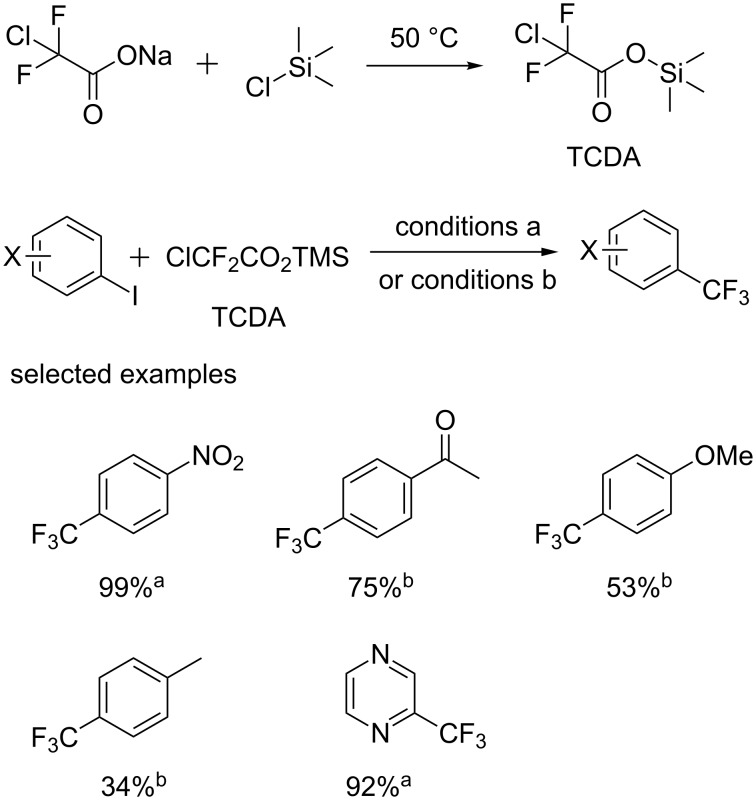
Trifluoromethylation of aryl iodides using TCDA as a trifluoromethyl source. Reaction conditions: [a] CuI (1.5 equiv), KF (5.0 equiv), TCDA (2.0 equiv) DMF/trimethylamine, 90 °C, 4 h, N_2_; [b] CuI (1.5 equiv), AgF (4.0 equiv), TMEDA (1.5 equiv), TCDA (2.0 equiv), DMF, 100 °C, 6 h.

Then, the group of Zhang [22] from GlaxoSmithKline designed trimethylsilyl chlorodifluoroacetate (TCDA) as a novel reagent, which was demonstrated to efficiently introduce a CF_3_ group via cooperative interaction of AgF and CuI (Scheme 10). In order to develop a practical process, the expensive AgF was replaced by KF. Under improved conditions, a broad range of aryl iodides were applicable to this protocol. The utility of this new reagent for the late-stage trifluoromethylation was demonstrated by the preparation of drug-related molecules, Boc-fluoxetine, fluvoxamine, and flutamide in decagram scale.

In 1989, the group of Chen [23] developed the first catalytic trifluoromethylation reaction of haloarenes with FSO_2_CF_2_CO_2_Me in the presence of CuI. It is proposed that the reaction pathway involved the formation of corresponding Cu(I)(O_2_CCF_2_SO_2_F). Later in 2016 [24], they sought to isolate and characterize the intermediate. During the study, they accidently prepared Cu(II)(O_2_CCF_2_SO_2_F)_2_ instead of the desired Cu(I)(O_2_CCF_2_SO_2_F), the former was demonstrated to be an efficient and mild trifluoromethylating reagent. It is a blue solid and can be conveniently prepared from inexpensive starting materials on a large scale.

**Scheme 11:**
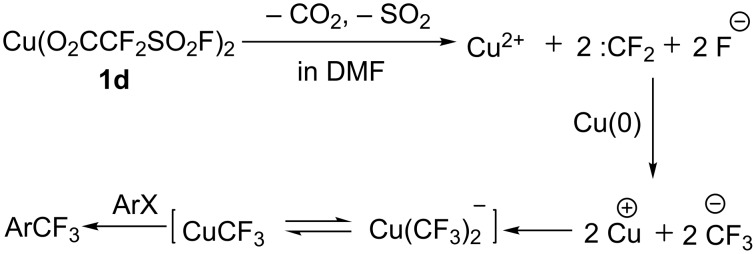
The mechanism of trifluoromethylation using Cu(II)(O_2_CCF_2_SO_2_F)_2_ as a trifluoromethyl source.

In this literature, the author proposed a plausible mechanism (Scheme 11). First, **1d** in DMF decomposed into Cu^2+^, difluorocarbene and fluoride with the release of SO_2_ and CO_2_. Then, difluorocarbene and fluoride combined into a CF_3_ species in the presence of Cu^2+^ and Cu. In contrast with the majority of previously reported copper-mediated trifluoromethylation reactions of haloarenes, CuCF_3_ generated in situ without any ligand reacts with the haloarenes to provide the corresponding products in good to excellent yields with good functional group compatibility.

### Copper-catalyzed trifluoromethylation of alkyl halides

Considerable efforts have been devoted to the trifluoromethylation of aryl halides in the past years. In contrast, successful examples of copper-catalyzed trifluoromethylation of alkyl halides are quite limited.

**Scheme 12:**
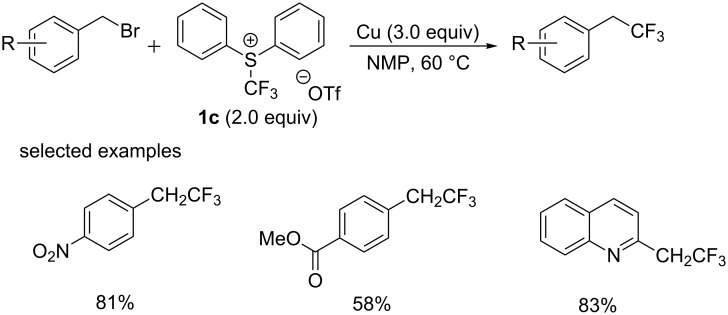
Trifluoromethylation of benzyl bromide reported by Shibata’s group.

In 2011, the group of Shibata [25] firstly reported the copper-mediated chemoselective trifluoromethylation at the benzylic position with shelf-stable electrophilic reagent **1c** (Scheme 12). In this protocol, benzyl bromides reacted with reactive [CuCF_3_] generated in situ from the reduction of **1c**. A broad range of benzyl bromides were found to be compatible with the reaction conditions, giving the corresponding products in good to high yields.

**Scheme 13:**
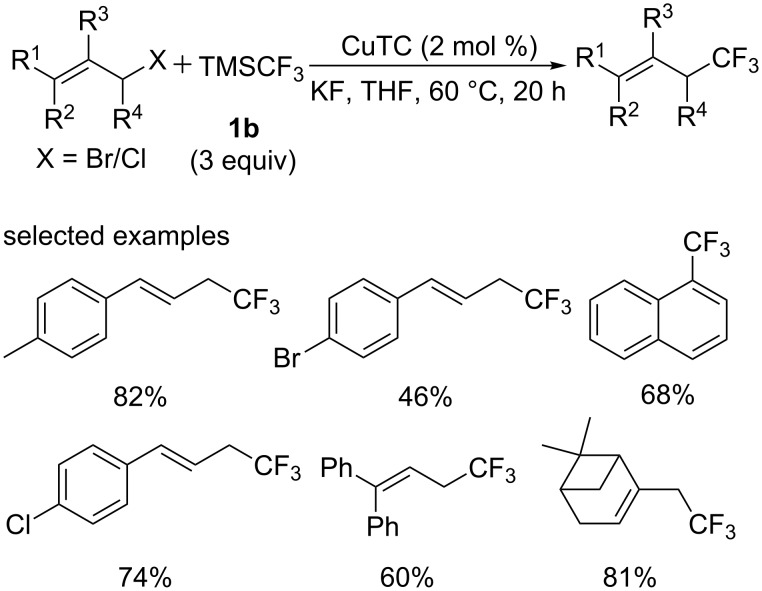
Trifluoromethylation of allylic halides and propargylic halides reported by the group of Nishibayashi.

Subsequently, a copper-catalyzed nucleophilic trifluoromethylation of allylic halides was developed by the group of Nishibayashi [26] (Scheme 13). Various cinnamyl halides bearing methyl, chloro, methoxy or ester groups proceeded smoothly to give the corresponding analogues in good yields. Other primary and secondary allylic halides were also subjected to these conditions. It is notable that the trifluoromethyl group was introduced at the α position of the carbon–halogen bond with complete regioselectivity.

**Scheme 14:**
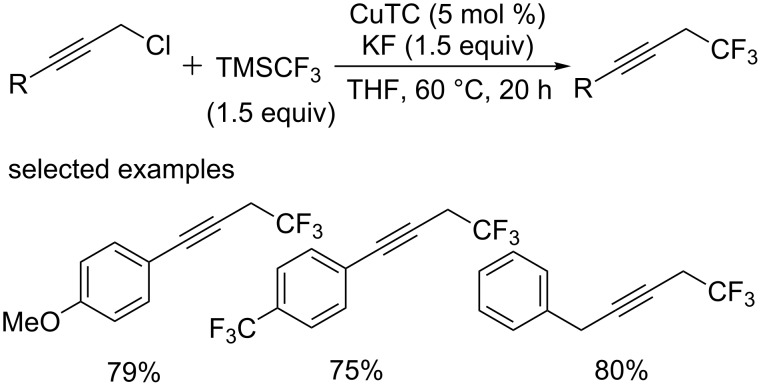
Trifluoromethylation of propargylic halides reported by the group of Nishibayashi.

Later, they extended these conditions to propargylic halides [27] and succeeded in synthesizing the corresponding analogues from primary propargylic chlorides (Scheme 14), while the trifluoromethylated allenes can be obtained from reactions of secondary propargylic chlorides.

**Scheme 15:**
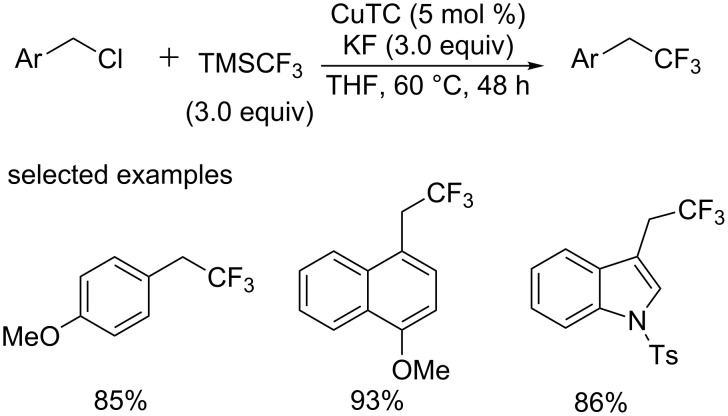
Trifluoromethylation of alkyl halides reported by Nishibayashi’s group.

Moreover, in 2014, the primary and secondary benzylic chlorides [28] were investigated under similar conditions, which proceeded smoothly to give the corresponding trifluoromethylated products in high yields (Scheme 15). But applicable substrates were limited to benzylic chlorides bearing electron-donating groups. The methodology described by the group of Nishibayashi could provide an efficient strategy for the synthesis of CF_3_-containing compounds at the allylic, propargylic, benzylic position, which were useful building blocks in pharmaceuticals.

Building on a pioneering work, substantial progress has recently been made in the trifluoromethylation of aryl and alkyl halides, including the development of new trifluoromethylation reagents or improved processes. But, there still exist several limitations in the trifluoromethylation of alkyl halides, such as limited substrate species and some unclarified mechanisms. The application of copper-catalyzed trifluoromethylation of alkyl halides remains in its infancy and has a lot of promise for the future.

### Copper-catalyzed trifluoromethylation of boronic acid derivatives

Boronic acid derivatives are common building blocks in organic chemistry due to its commercial availability, and stability to heat, air, and water. Several examples of trifluoromethylation of boronic acid derivatives with nucleophilic, electrophilic or radical trifluoromethylation reagents were reported.

#### Trifluoromethylation of boronic acid derivatives with nucleophilic trifluoromethylation reagents

**Scheme 16:**
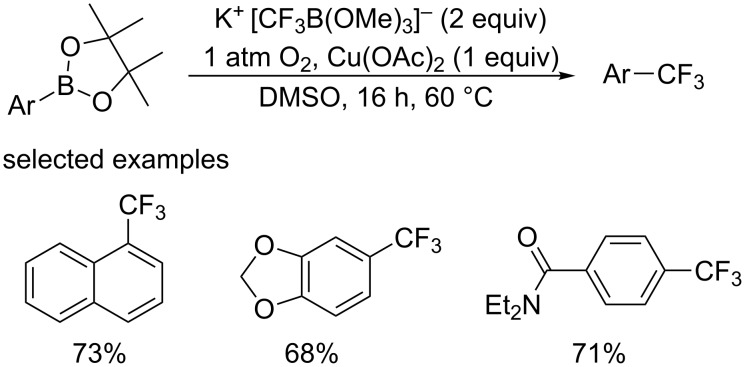
Trifluoromethylation of pinacol esters reported by the group of Gooßen.

Protodeborylation was found to be the main side reaction in the copper-catalyzed trifluoromethylation of arylboronic acids [29–30]. In 2012, the group of Gooßen [31] designed a new protocol replacing the boronic acids with the corresponding pinacol esters to minimize side reaction (Scheme 16). This reaction proceeded smoothly to form the corresponding analogues in moderate to high yields using the shelf-stable, and easy-to-handle potassium (trifluoromethyl)trimethylborate K^+^[CF_3_B(OMe)_3_]^−^ as a CF_3_ source, molecular oxygen as the oxidant. However, another side reaction, substitution of the boronate by methoxy groups originating from the CF_3_ source, arose in this transformation.

**Scheme 17:**
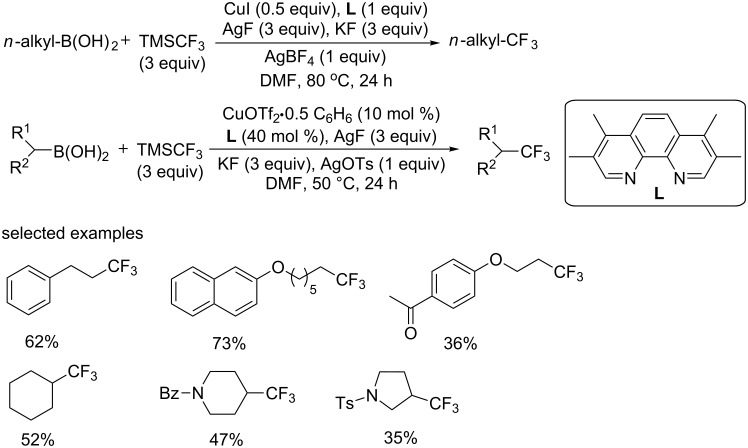
Trifluoromethylation of primary and secondary alkylboronic acids reported by the group of Fu.

At the same year, the group of Fu explored the trifluoromethylation of primary and secondary alkylboronic acids with the Ruppert–Prakash reagent (TMSCF_3_) (Scheme 17) [32]. These alkylboronic acids were prepared from the corresponding alkyl halides or tosylates by using their previously developed Cu-catalyzed borylation method [33]. Both primary and secondary alkylboronic acids underwent the trifluoromethylation well under different optimized conditions.

#### Trifluoromethylation of boronic acid derivatives with electrophilic trifluoromethylation reagents

**Scheme 18:**
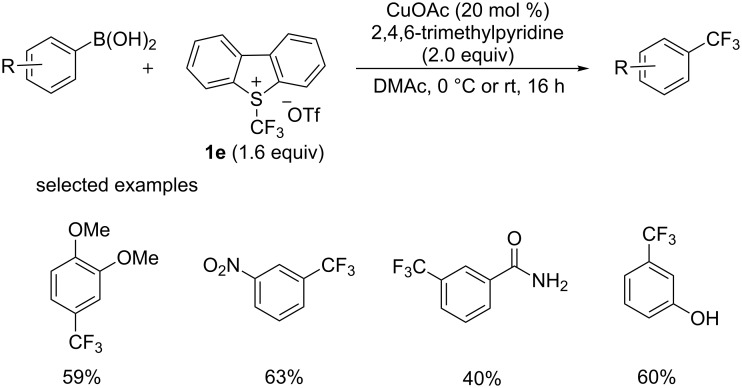
Trifluoromethylation of boronic acid derivatives reported by the group of Liu.

CF_3_^+^ reagents were also explored in the trifluoromethylation of boronic acid derivatives. In 2011, the group of Liu [34] achieved the copper-catalyzed trifluoromethylation of aryl, heteroaryl, and vinylboronic acids at room temperature or 0 °C with Umemoto’s reagent **1e** (Scheme 18). Of importance, this process was not sensitive to moisture, unlike the former trifluoromethylation reaction involved CF_3_^−^ reagents. Moreover, this process showed good functional group compatibility and even some unprotected OH and NH groups were tolerated.

**Scheme 19:**
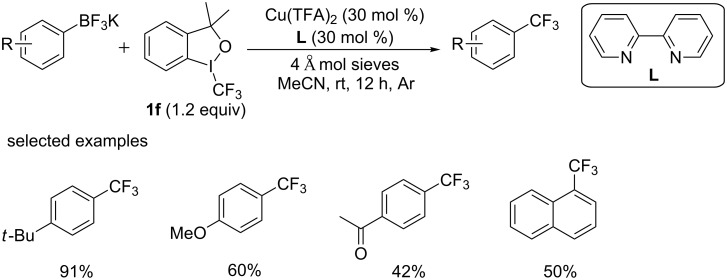
Trifluoromethylation of organotrifluoroborates reported by the group of Huang.

Subsequently, the group of Huang [35] reported an efficient copper-catalyzed trifluoromethylation of organotrifluoroborates (Scheme 19). This reaction was accomplished under base-free conditions by using Togni’s reagent **1f** as the trifluoromethylation reagent at room temperature. Organotrifluoroborates were attractive alternatives to boronic acids for its superior characteristic, such as ease of handling, storability, and robustness under harsh reaction conditions. It was found that ligands and molecular sieves were essential for the efficient conversion. Various aryltrifluoroborates bearing electron-donating and electron-withdrawing substituents at different positions were converted to the desired products. And substrates bearing electron-withdrawing groups suffered from lower yields.

**Scheme 20:**
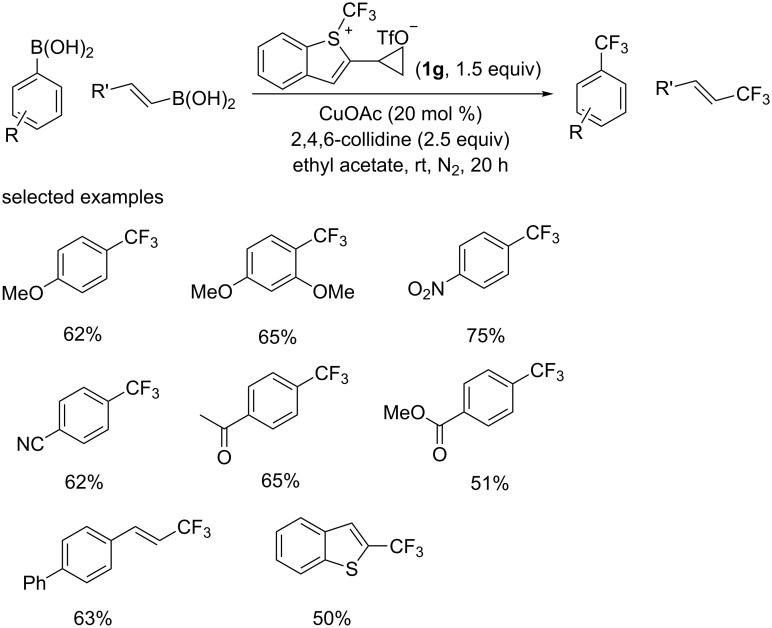
Trifluoromethylation of aryl- and vinylboronic acids reported by the group of Shibata.

Recently, the group of Shibata [36] reported a catalytic trifluoromethylation of aryl- and vinylboronic acids using **1g** as a trifluoromethyl source (Scheme 20), which had not been actively investigated due to its instability in some solvents [37]. Solvent screening results indicated that ethyl acetate was the best solvent for the target transformations. Substrates regardless of bearing electron-donating (OMe) or electron-withdrawing groups (NO_2_, CN, carbonyl, and ester) were tolerated in this conversion. Moreover, vinylboronic acids and heteroaryl substrates were also acceptable.

#### Trifluoromethylation of boronic acid derivatives with radical trifluoromethylation reagents

The trifluoromethylation of boronic acid derivatives through a radical pathway was explored since the CF_3_ radical can be generated under mild, neutral conditions from commercially available and relatively inexpensive CF_3_I. Besides, the easy conversion of CF_3_I to CF_3_ radical at room temperature with visible light developed by MacMillan facilitated these method [38–39].

**Scheme 21:**
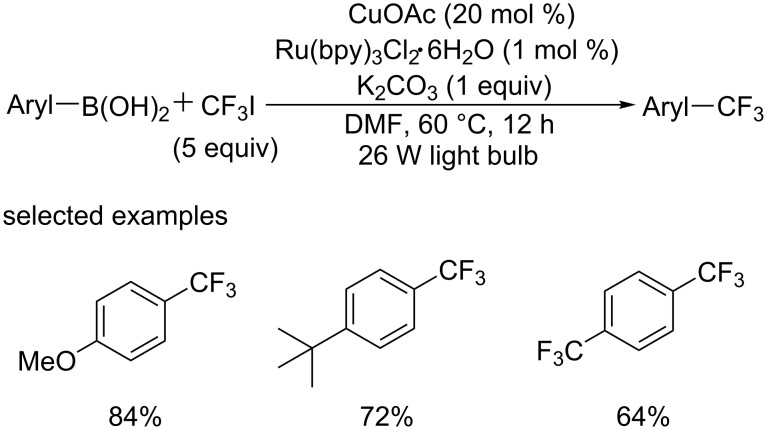
Trifluoromethylation of arylboronic acids via the merger of photoredox and Cu catalysis.

On the basis of the former work, the group of Sanford [40] designed the cross-coupling of arylboronic acids with CF_3_I via the merger of photoredox and Cu catalysis (Scheme 21). In this protocol, the CF_3_ radical was generated by visible light photoredox, then Cu aryl species were generated through Cu catalysis. Aromatic boronic acids bearing either electron-donating or electron-withdrawing substituents underwent trifluoromethylation smoothly to give the corresponding products in high yield. It represented a new example of combining transition metal and photoredox catalysis to achieve the trifluoromethylation of (hetero)aromatic boronic acids.

**Scheme 22:**
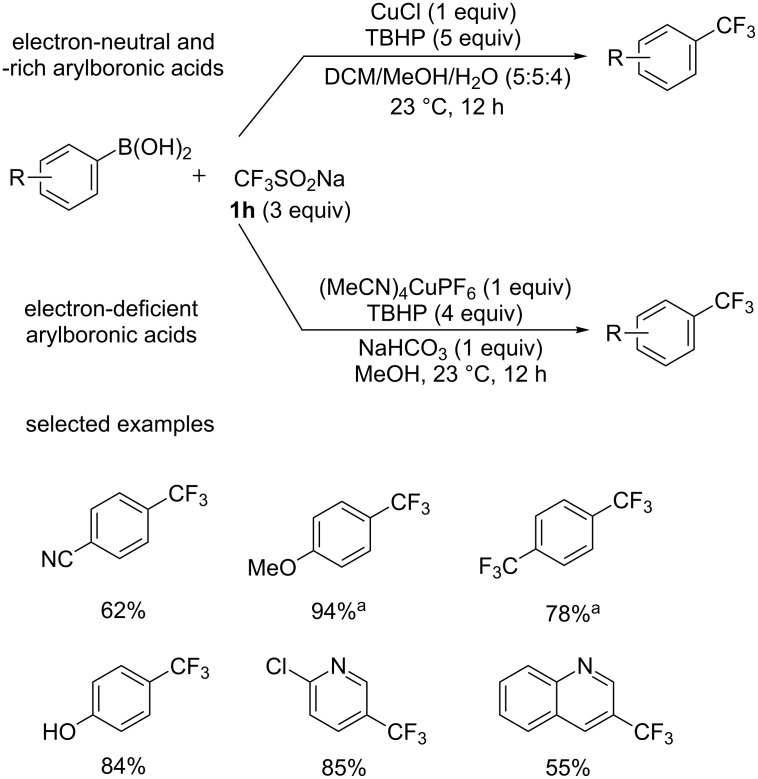
Trifluoromethylation of arylboronic acids reported by Sanford’s group. Isolated yield. ^a^Yields determined by ^19^F NMR analysis.

Then, the same group [41] chose a more practical CF_3_ source, CF_3_SO_2_Na (Langlois’ reagent, **1h**, Scheme 22), which can generate trifluoromethyl radicals at room temperature in the presence of ambient air and moisture when combined with TBHP. Electron-neutral and -rich boronic acids proceeded smoothly to give the corresponding products in excellent yields. An addition of NaHCO_3_ was required for the conversion of electron-deficient derivatives. Besides, sterically hindered boronic acids were also tolerated in this reaction. This protocol was simplified and easy-to-handle and no protodeboronation byproduct was observed under these conditions, while the major side product (the corresponding hydroxylated arene) was readily removable by extraction or column chromatography.

**Scheme 23:**
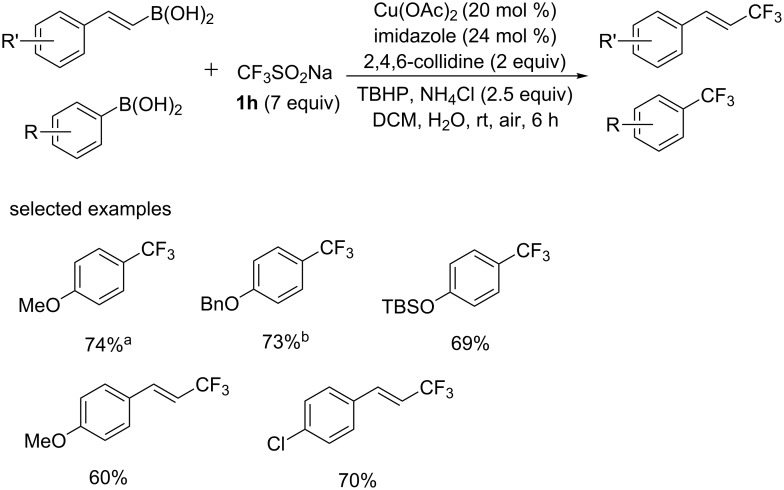
Trifluoromethylation of arylboronic acids and vinylboronic acids reported by the group of Beller. Yields determined by ^19^F NMR analysis. ^a^GC yield. ^b^Isolated yield.

Later, the group of Beller [42] described another example of copper-catalyzed trifluoromethylation reactions of aryl- and vinylboronic acids with CF_3_SO_2_Na as the trifluoromethyl source (Scheme 23). The trifluoromethyl radical was generated from CF_3_SO_2_Na in the presence of TBHP at room temperature using a mixture of water and DCM as solvent. Arylboronic acids with electron-donating substituents proceeded smoothly to give the corresponding products in good yields. Common hydroxy protecting groups (Bn and TBS) were well-tolerated in this process. Also, the vinylboronic acids were compatible with the reaction, which were less sensitive towards the influence of the substituents.

Great advances have been made in the trifluoromethylation of boronic acid derivatives. However, some side reaction existed and the substrate scope was still need to be expanded. Therefore, more efficient and practical methods were desirable.

#### Trifluoromethylation of aryldiazonium salts (Sandmeyer type trifluoromethylation)

Aromatic amines, which are available in great structural diversity, are prevalent chemicals in organic chemistry. The amino group can be easily transformed into numerous functional groups including halides or cyano groups, which is known as the Sandmeyer reaction. This transformation contained two steps: Diazotization of aromatic amines led to aryldiazonium salt, followed by conversion of diazonium group into target functional groups. Recently, several examples of conversion of anilines into trifluoromethylated arenes, named Sandmeyer type trifluoromethylation, were disclosed. This protocol offered a complementary method for the synthesis of trifluoromethylated arenes from the corresponding aromatic amines.

**Scheme 24:**
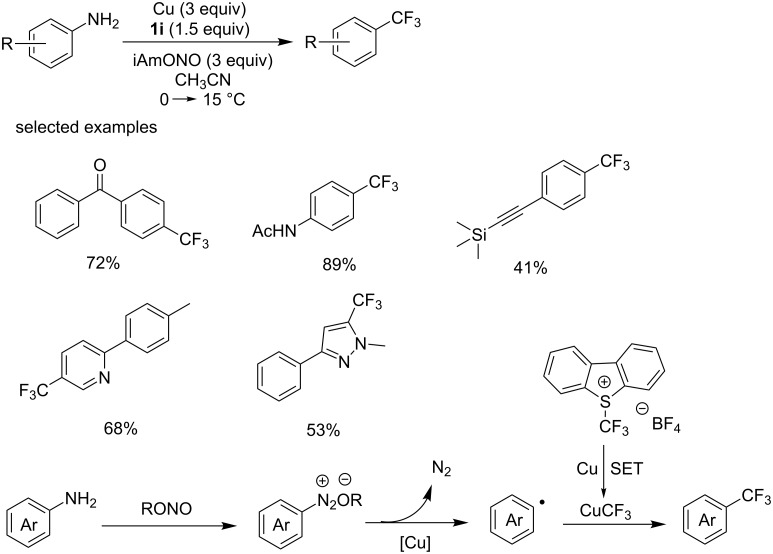
Copper-mediated Sandmeyer type trifluoromethylation using Umemoto’s reagent as a trifluoromethylation reagent and the proposed mechanism.

In 2013, Fu [43], Gooßen [44] and Wang [45] almost simultaneously reported Sandmeyer type trifluoromethylations. The group of Fu [43] accomplished a copper-mediated Sandmeyer trifluoromethylation reaction for the conversion of aromatic amines into trifluoromethylated arenes (Scheme 24). This reaction was conducted under mild conditions using Umemoto’s reagent as the trifluoromethylation agent in the presence of isoamyl nitrite (iAmONO). The reaction proceeded smoothly with various arylamines under these conditions to give the corresponding products in modest to good yields. The reaction exhibits good tolerance to many functional groups, such as unsaturated double bonds, triple bonds and even unprotected OH group. Different heteroaromatic amines were also amenable to this conversion, including pyridines and pyrazoles.

A mechanistic study indicated that an aryl radical and CuCF_3_ were involved in this conversion. A plausible mechanism is proposed in Scheme 24. First, the CF_3_ radical, generated from Umemoto’s reagent through copper-mediated single electron transfer (SET), reacts with copper affording CuCF_3_. Second, Ar–CF_3_ was formed by the reaction of CuCF_3_ with the aryl radical derived from the aryldiazonium ion, which is generated in situ from the corresponding arylamine.

**Scheme 25:**
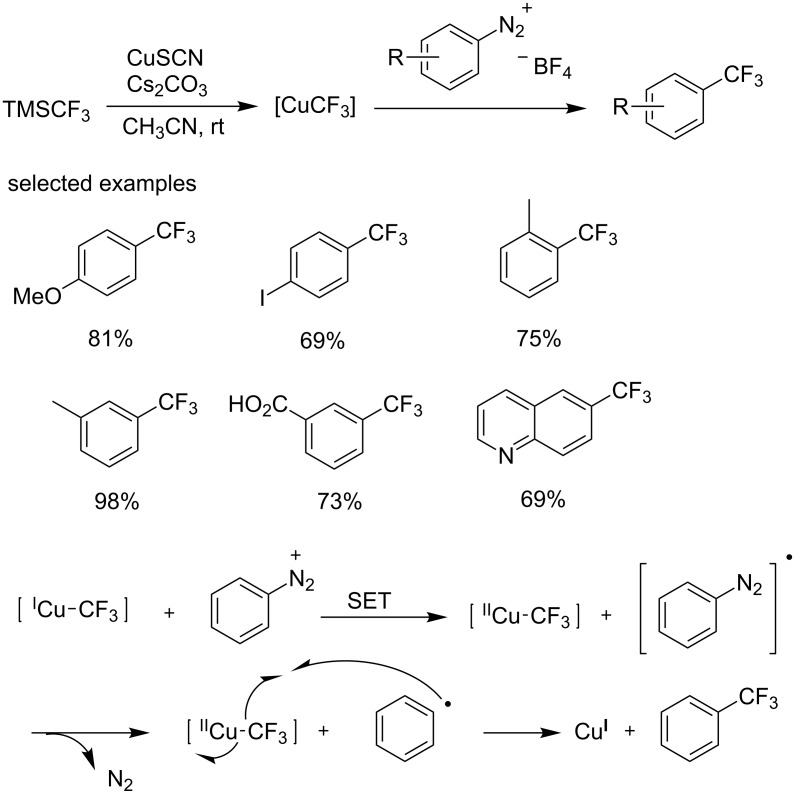
Copper-mediated Sandmeyer type trifluoromethylation using TMSCF_3_ as a trifluoromethylation reagent and the proposed mechanism.

At the same time, a copper-catalyzed Sandmeyer trifluoromethylation of arenediazonium tetrafluoroborates was also reported by the group of Gooßen (Scheme 25) [44]. This reaction was conducted under mild conditions using TMSCF_3_ as the trifluoromethyl source. It is notable that no desired product was observed in the absence of copper or basic additives. Both electron-rich and electron-deficient substrates were smoothly converted into the corresponding analogues in high yields except for some simple, low-boiling substrates. Additionally, various heterocycles were also compatible with this approach. The reaction can tolerate many common groups, including methoxy, iodo and carboxyl groups. The majority of products were produced in sufficiently pure form to permit straightforward isolation.

Gooßen assumed that this conversion proceeded via radical intermediates (Scheme 25), which is analogous to Sandmeyer halogenations of diazonium salts. First, the trifluoromethyl copper(I) species is generated from TMSCF_3_ and copper salt. Then, Cu(I)CF_3_ transfers one electron to the diazonium salt affording Cu(II)CF_3_ and a diazo radical following by the formation of an aryl radial from the diazo radical through the release of nitrogen. Finally, the aryl radical reacts with Cu(II)CF_3_ furnishing the trifluoromethylated product along with copper(I) salt.

**Scheme 26:**
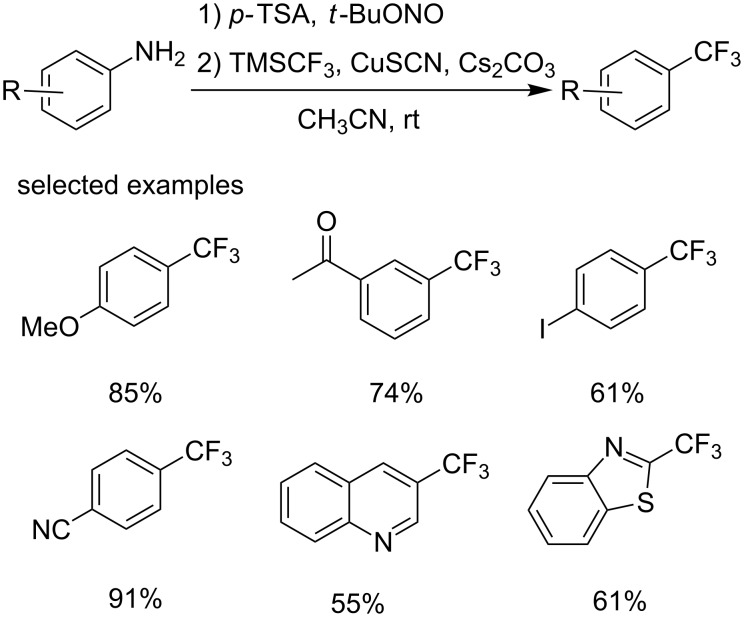
One-pot Sandmeyer trifluoromethylation reported by the group of Gooßen.

Compared with Fu’s method, this process needs an extra step to generate the diazionium salt. Later, the same group developed a one-pot Sandmeyer trifluoromethylation combining the diazotization and the Sandmeyer reaction (Scheme 26) [46]. This reaction proceeded smoothly using *tert*-butyl nitrite as the diazotization reagent, TMSCF_3_ as the trifluoromethylation reagent in the presence of anhydrous *para*-toluenesulfonic acid and catalytic amounts of copper reagent (0.5 equiv). Diversely substituted aromatic amines and heterocyclic amines were all converted in high yields. The majority of products were produced in sufficiently pure form to allow for simple isolation.

**Scheme 27:**
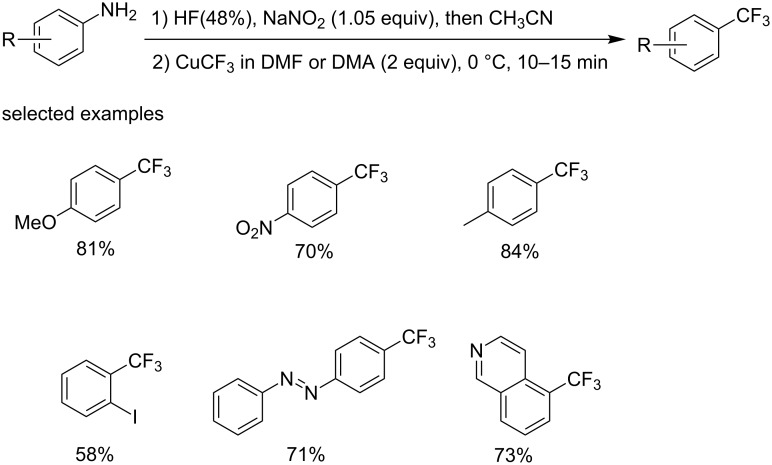
Copper-catalyzed trifluoromethylation of arenediazonium salts in aqueous media.

Note that the CF_3_ reagents employed in the aformentioned examples were all moisture sensitive. Therefore, it required anhydrous conditions, which limits the large-scale application. In order to overcome this problem, the group of Grushin [47] developed a copper-catalyzed trifluoromethylation of arenediazonium salts in aqueous media (Scheme 27).

Initially, they investigated the trifluoromethylation of preisolated arenediazonium salts 4-XC_6_H_4_N_2_^+^ BF_4_^−^ (X = MeO, Br, NO_2_). But the main product was the reduction product XC_6_H_5_, which could be largely suppressed using MeCN as a co-solvent. Then, they examined the reaction of CuCF_3_ with ArN_2_^+^ X^−^ generated in situ from the corresponding aniline. It was found that *t*-BuONO and aromatic amines decomposed CuCF_3_. Acid was added to make the diazonium substrate fully preformed prior to the reaction with CuCF_3_. Finally, they explored the possibility of reaction of hydrolysable CuCF_3_ reagent with aqueous solutions of arenediazonium salts. Although it seemed unwise, the desired ArCF_3_ product was produced. Subsequent condition optimization provided a high-yielding process. The reaction can tolerate a variety of synthetically important functional groups, such as Me, MeO, NO_2_, I. A mechanistic study revealed that a radical mechanism was involved in this transformation.

**Scheme 28:**
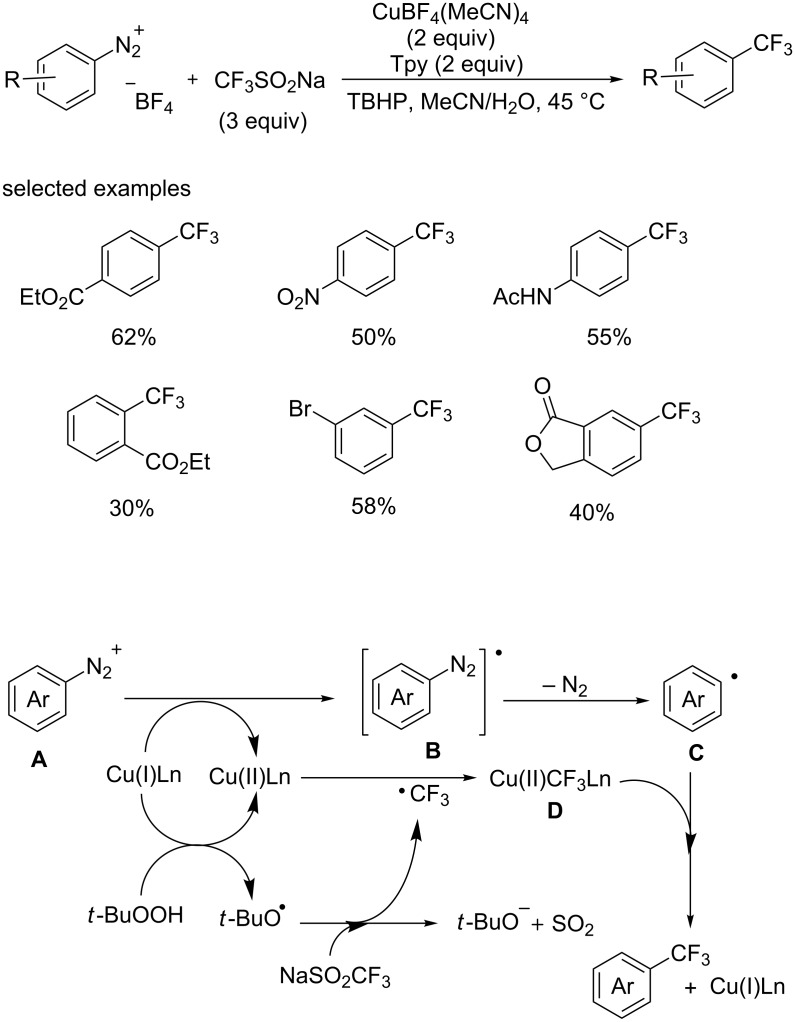
Copper-mediated Sandmeyer trifluoromethylation using Langlois’ reagent as a trifluoromethyl source and the proposed mechanism.

Recently, the group of Qing [48] accomplished a Sandmeyer trifluoromethylation of aryldiazonium derivatives with NaSO_2_CF_3_ in the presence of TBHP (Scheme 28). The yield was improved by adding the tridentate ligand 2,2′,6′,2″-terpyridine (tpy) and a small amount of water as the co-solvent. A range of arenediazonium tetrafluoroborates were smoothly converted to the corresponding analogues in acceptable yields.

A mechanistic study indicated that a radical process was involved in this conversion (Scheme 28). First, the diazo radical, generated through copper-mediated single electron transfer from diazonium salt **A**, released nitrogen gas affording the aryl radical **C**. On the other hand, the CF_3_ radical was generated through the reaction of TBHP with NaSO_2_CF_3_ in the presence of Cu(I) species. Then, the CF_3_ radical reacted with the Cu(I) species to provide the complex Cu(II)CF_3_Ln. Finally, the reaction of aryl radical **C** with complex **D** gave the desired Ar–CF_3_ product along with the Cu(I) species.

### Copper-catalyzed direct trifluoromethylation of C–H bonds

The above-mentioned strategies to introduce a trifluoromethyl group into organic compound were based on the use of prefunctionalized substrates. Instead, the direct trifluoromethylation of C–H bonds of arenes and heteroarenes was a more efficient and ideal protocol due to its atom and step economy. However, the direct trifluoromethylation of C–H bonds was not simple. And only in recent years, extensive research was reported including direct trifluoromethylation of C(sp^3^)–H, C(sp^2^)–H, C(sp)–H bonds in various substrates.

#### Copper-catalyzed direct trifluoromethylation of C(sp^3^)–H

Molecules containing allylic CF_3_ functional groups are versatile precursors for the synthesis of different types of CF_3_-containing molecules. Traditional methods that were described for the preparation of CF_3_-containing molecules required harsh reaction conditions and pre-functionalized starting materials such as allyl bromides or fluorosulfones. The past years had witnessed many advances in constructing allylic CF_3_ bonds from olefins. Following examples described the straightforward trifluoromethylation of terminal alkenes via allylic C(sp^3^)–H bond activation generating allylic trifluoromethylated compounds.

**Scheme 29:**
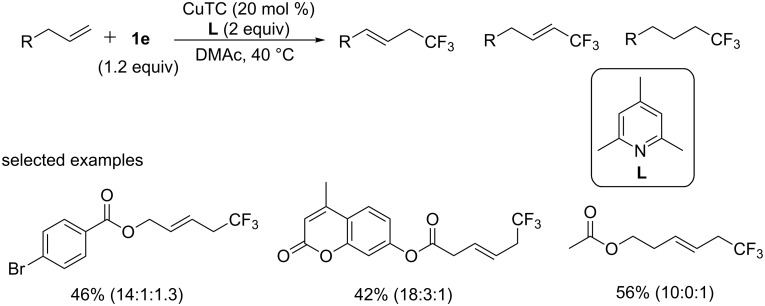
Trifluoromethylation of terminal alkenes reported by the group of Liu.

In 2011, the group of Fu and Liu [49] described an unprecedented type of a Cu-catalyzed trifluoromethylation reaction of terminal alkenes through C(sp^3^)–H activation (Scheme 29). Many substrates underwent the trifluoromethylation smoothly using Umemoto’s reagent **1e** as a trifluoromethyl source and CuTC as catalyst under mild conditions. This reaction was tolerant to moisture and was compatible to different functional groups. But the reaction was sensitive to the steric hindrance of the olefin substrates, and 2-substituted terminal alkenes or internal (including cyclic) alkenes were not applicable to this protocol. A mechanistic study showed that the reaction may proceed through a Heck-like four-membered-ring transition state. Note that the presence of an olefin moiety in the product promised further conversion to other types of CF_3_-containing molecules.

**Scheme 30:**
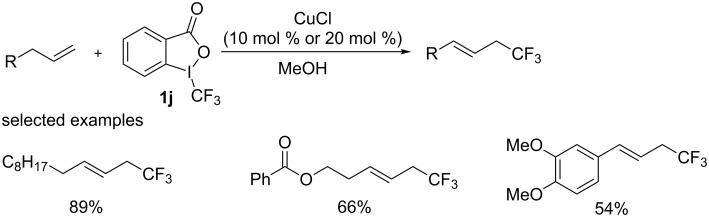
Trifluoromethylation of terminal alkenes reported by the group of Wang.

Later, the group of Wang [50] employed cheap copper chloride as the catalyst and a hypervalent iodine(III) reagent **1j** as both the oxidant and the CF_3_ source in allylic trifluoromethylation (Scheme 30). It was found that allyl substrates bearing an aromatic moiety showed relatively low efficiency.

**Scheme 31:**
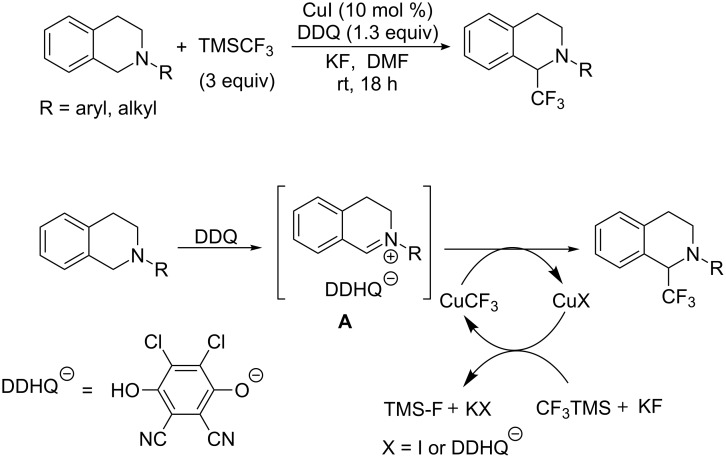
Trifluoromethylation of tetrahydroisoquinoline derivatives reported by Li and the proposed mechanism.

At the same year, the group of Li [51] achieved a copper-catalyzed trifluoromethylation via oxidation of C(sp^3^)–H bonds adjacent to nitrogen atom in tetrahydroisoquinoline derivatives using DDQ and Ruppert–Prakash reagent (Scheme 31). A variety of amines proceeded smoothly to give the corresponding products in 15–90% yields under mild conditions.

Based on previous literature, the author proposed a possible mechanism in Scheme 31. Firstly, oxidation of *N*-substituted tetrahydroisoquinoline with DDQ generates dihydroquinoline salt **A**. Next, CuCF_3_, generated by the reaction of CuI and CF_3_TMS/KF, undergoes a nucleophilic addition with **A** affording the desired products and the copper salt. The generated copper salt would be reused to form CuCF_3_ in the nucleophilic step again. So, only a catalytic amount of copper salt was required in this reaction.

The hydrogens on the *ortho* and *para* positions of phenols have higher reactivity. Thus, undesired side reactions were often involved in the trifluoromethylation of less substituted phenols, including oxidative dimerization and oligomerization. Although phenols were widely used building blocks in bioactive compounds, only a few examples introducing a CF_3_ have been reported.

**Scheme 32:**
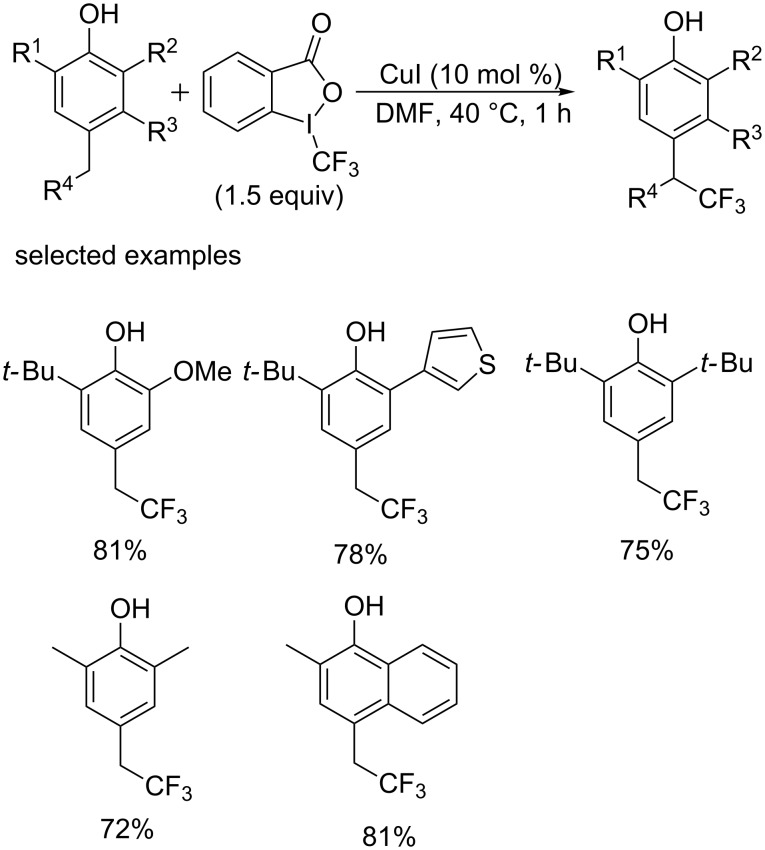
Trifluoromethylation of phenol derivatives reported by the group of Hamashima.

In 2015, the group of Hamashima [52] achieved a direct C–H trifluoromethylation of phenol derivatives with high regioselectivity. The CF_3_ was selectively incorporated into the *para*-benzylic position of the hydroxy group (Scheme 32). It is notable that the bulky *tert*-butyl group was introduced to suppress side reactions, which could be removed under acidic conditions. The solvent was critical in terms of product switching; the trifluoromethylation of aromatic C–H occurred in alcoholic solvents. The author accomplished the synthesis of a potent enoyl-acyl carrier protein reductase (Fab I) inhibitor in high yield under this conditions, demonstrating the practical utility of this process.

#### Copper-catalyzed direct trifluoromethylation of C(sp^2^)–H

**Scheme 33:**
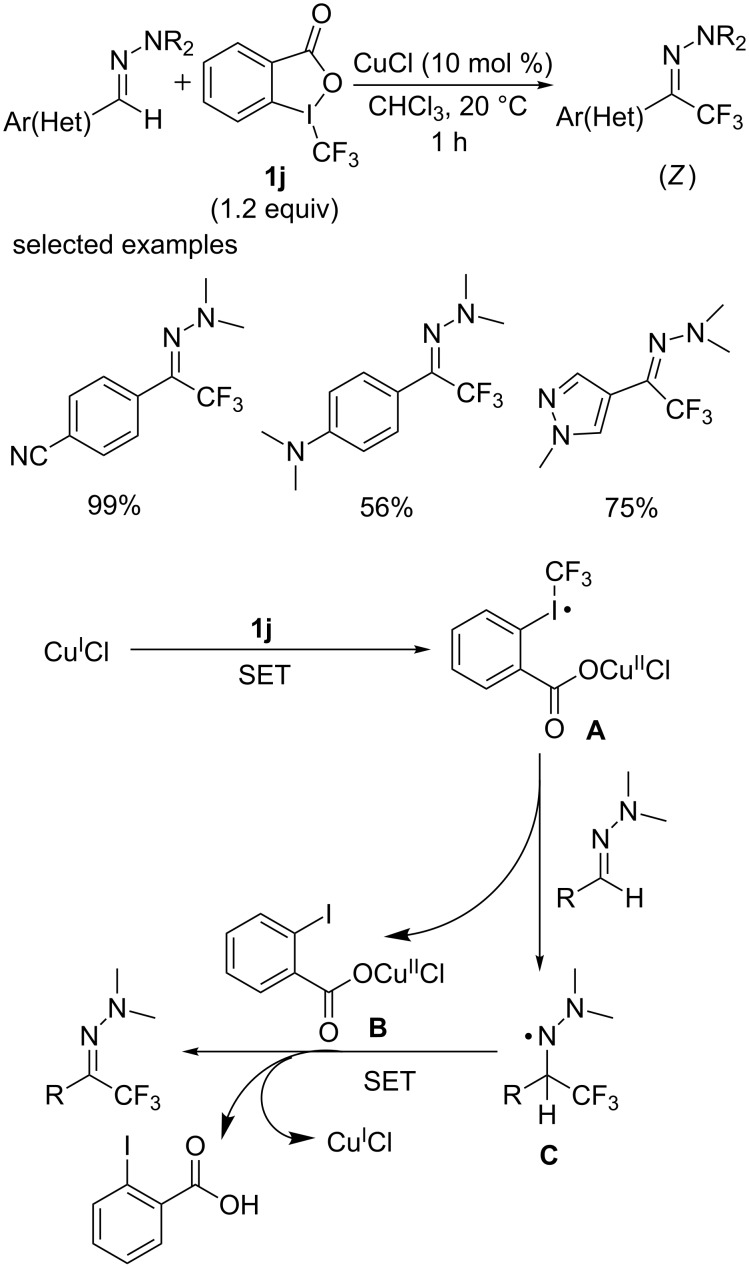
Trifluoromethylation of hydrazones reported by the group of Baudoin and the proposed mechanism.

**Direct trifluoromethylation of C(sp^2^)–H with a electrophilic trifluoromethylation reagent (Togni’s reagent):*** N,N*-Dialkylhydrazones were widely used in organic chemistry, including using as synthetic equivalents of carbonyl compounds, as precursors to substituted hydrazines or primary amines. In 2013, Baudoin and co-workers [53] firstly achieved the incorporation of CF_3_ into *N,N*-dialkylhydrazones using Togni’s reagent as a trifluoromethyl source in the presence of simple copper chloride (Scheme 33). Various hydrazones with an electron-donating dialkylamino group including 1-piperidinyl and 4-morpholinyl participated efficiently in this reaction. Irrespective of the phenyl group was substituted by an electron-withdrawing or -donating group, the reaction proceeded smoothly to afford the desired products in high yields. Besides, several heterocyclic hydrazones including pyridinyl, pyrazolyl and furyl were also applicable under this conditions. It is of importance, that the trifluoromethylated hydrazones were formed exclusively as the *Z* isomers.

The proposed mechanism [53] is shown in Scheme 33. Activation of Togni’s reagent by CuI through single-electron transfer (SET) initiates the reaction pathway to generate the CF_3_ radical donor, copper(II) species **A**. The latter reacts with the hydrazone to the trifluoromethylated aminyl radical intermediate **C** which is stabilized by the lone pair of the adjacent nitrogen atom, and (2-iodobenzoyloxy)copper(II) chloride (**B**). Finally, intermediate **C** is oxidized by copper(II) to restore the hydrazone functional group and copper(I).

**Scheme 34:**
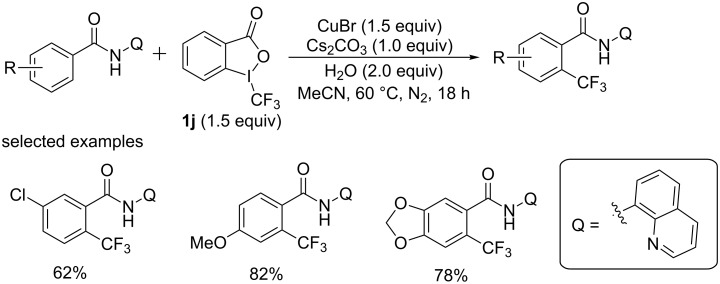
Trifluoromethylation of benzamides reported by the group of Tan.

Benzamides were widely used building blocks in medicinal chemistry. In 2014, Dai and Yu [54] achieved an elegant copper-mediated *ortho*-selective trifluoromethylation of benzamides assisted by an *N*-phenyloxazoline group. But one major problem was the low selectivity, and that the ditrifluoromethylation product was formed as well. Recently, Tan and co-workers [55] designed a highly mono-selective *ortho*-trifluoromethylation of benzamides assisted with an 8-aminoquinoline directing group. This reaction employed simple copper salt CuBr as the promoter and Togni’s reagent II as a CF_3_ source (Scheme 34). Addition of water benefited the yield. A variety of functional groups such as methyl, ethyl, ester, fluoro, chloro, bromo, phenyl, methoxy, ethoxy as well as trifluoromethyl groups were well tolerated on the phenyl ring of benzamides and various *ortho*-trifluoromethylated benzamides were efficiently synthesized in 36–82% yields.

**Scheme 35:**
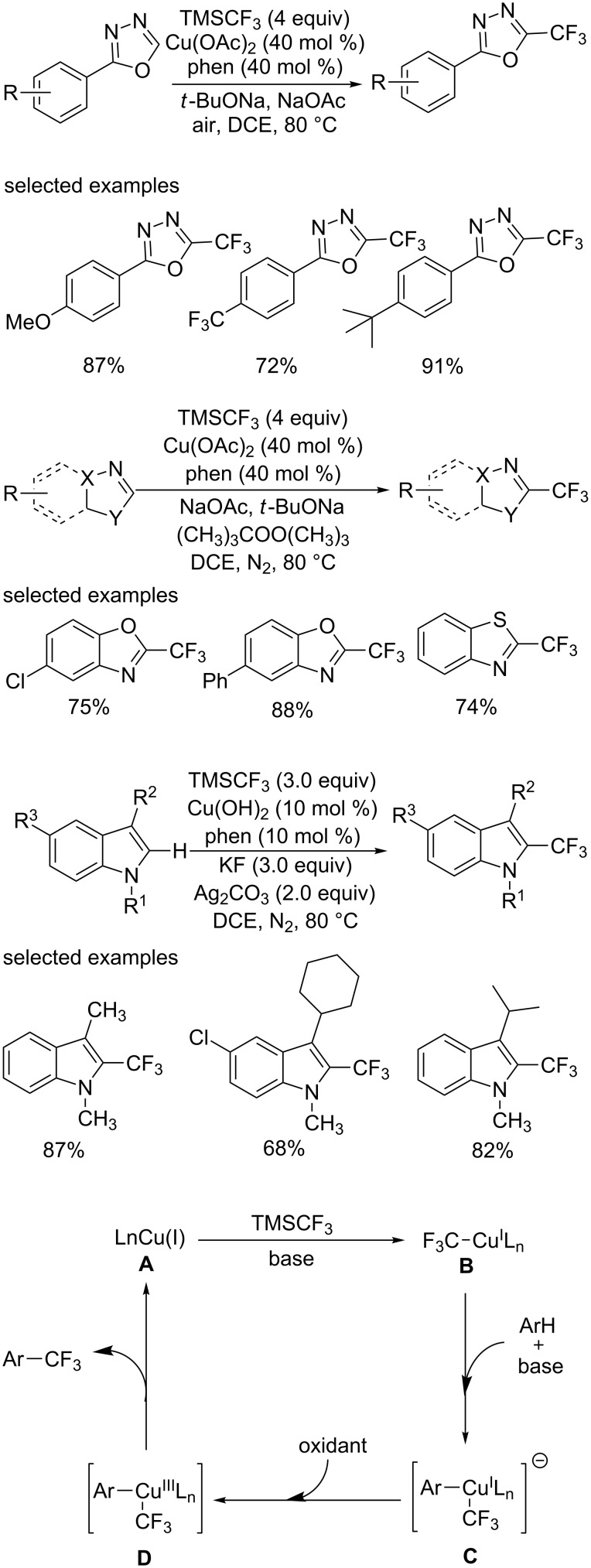
Trifluoromethylation of heteroarenes and electron-deficient arenes reported by the group of Qing and the proposed mechanism.

**Direct trifluoromethylation of C(sp^2^)–H with a nucleophilic trifluoromethylation reagent (TMSCF_3_):** Previously, the radical and electrophilic trifluoromethylation of arenes and heteroarenes were often limited to substrates bearing electron-donating substituents and generate mixture of regioisomers in some cases. In 2012, the group of Qing [56] designed a copper-catalyzed oxidative trifluoromethylation of heteroarenes and electron-deficient arenes with TMSCF_3_ through direct C−H activation (Scheme 35).

At first, the oxidative trifluoromethylation of 1,3,4-oxadiazoles proceeded smoothly using TMSCF_3_ as a trifluoromethyl source and air as an oxidant to give the corresponding products in high yields. Various 1,3,4-oxadiazoles bearing electron-donating and electron-withdrawing groups at the *para* position on the aryl rings were well tolerated, although the latter showed lower efficiency.

Then, they extended the substrate scope to 1,3-azoles and perfluoroarenes. Di-*tert*-butyl peroxide was chosen as a suitable oxidant instead of air. Functional groups, such as chloro and bromo, were compatible in this reaction, providing a complementary platform for further conversion. Notably, electron-deficient pentafluorobenzene was highly reactive under these reaction conditions to afford octafluorotoluene in excellent yield, which provided a promising model for functionalizations of electron-deficient arenes.

Furthermore, electron-rich indoles were applicable in these conditions and Cu(OH)_2_ and Ag_2_CO_3_ were the best catalyst and oxidant, respectively. The electron density of the pyrrole ring had impact on the efficiency. *N*-Tosylindole and indole bearing the CO_2_Me group on C3 position were nearly unreactive.

A plausible mechanism was proposed based on preliminary mechanistic studies (Scheme 35). First, the key intermediate CF_3_Cu^I^Ln is generated in situ by the reaction of TMSCF_3_ with Cu(II) reagent, followed by transmetalation with activated Ar−H generating the (aryl)Cu^I^(CF_3_) species **C**, which might be oxidized to the corresponding (aryl)Cu^III^(CF_3_) intermediate **D**. Finally, reductive elimination of the intermediate **D** would afford the desired product and regenerate Cu(I) catalyst to restart the catalytic cycle.

**Direct trifluoromethylation of C(sp^2^)–H with a radical trifluoromethylation reagent (CF_3_SO_2_Na):** The radical trifluoromethylation via direct C(sp^2^)–H activation also have made significant progress in recent years. Among them, the most studied methods were trifluoromethylations using Langlois’ reagent (CF_3_SO_2_Na) as the trifluoromethyl source, which was inexpensive and readily available.

**Scheme 36:**
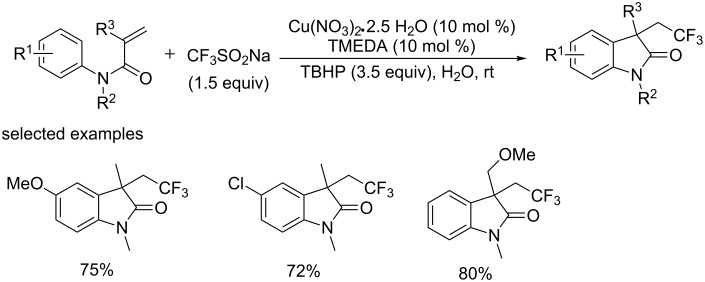
Trifluoromethylation of *N*-aryl acrylamides using CF_3_SO_2_Na as a trifluoromethyl source.

In 2014, the group of Liang and Lipshutz [57] explored a copper-catalyzed trifluoromethylation of *N*-aryl acrylamides using Langlois’ reagent (CF_3_SO_2_Na) as a trifluoromethyl source and water as the reaction medium (Scheme 36). A variety of CF_3_-containing oxindoles bearing a quaternary carbon center were formed under this conditions. Furthermore, more CF_3_SO_2_Na (3.0 equiv) and TBHP (7.0 equiv) were employed by highly water-insoluble solid substrates and substrates bearing electron-withdrawing groups.

This protocol exhibited several noteworthy features, such as the inexpensive and readily available catalyst and the trifluoromethylation reagent, and the ease of handing all components in air, the environmentally friendly nature, and the recycling of the aqueous medium.

**Scheme 37:**
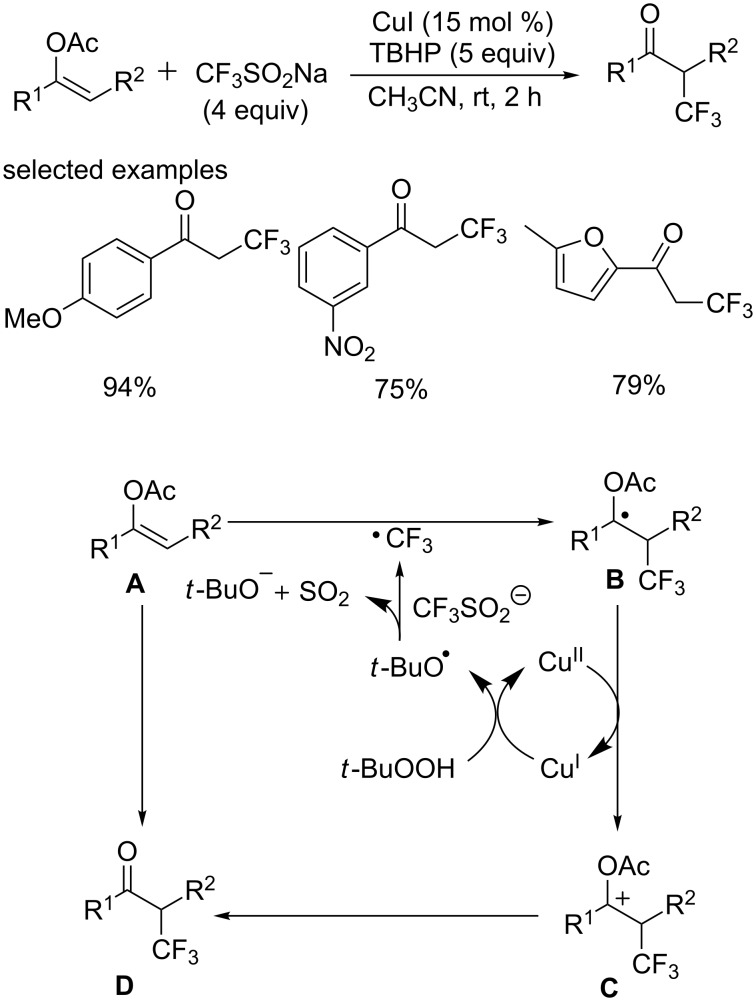
Trifluoromethylation of aryl(heteroaryl)enol acetates using CF_3_SO_2_Na as the source of CF_3_ and the proposed mechanism.

Subsequently, the group of Li and Duan [58] reported an efficient method for the synthesis of α-trifluoromethyl ketones via addition of CF_3_ to aryl(heteroaryl)enol acetates using readily available CF_3_SO_2_Na (Scheme 37). This reaction was experimentally simple and set out at room temperature under ambient conditions, affording the corresponding products in good to excellent yields with wide functional group tolerance.

The mechanism research suggested that the CF_3_ radical was involved in this transformation (Scheme 37). Firstly, the trifluoromethyl radical was generated in situ by the reaction of *tert*-butyl hydroperoxide with CF_3_SO_2_Na in the presence of catalytic amounts of CuI. Next, the addition of the CF_3_ radical to the electron-rich α-position of the substrates formed the radical species **B**. Subsequent oxidation by Cu(II) produced cationic intermediate **C** and regenerated Cu(I) to restart the catalytic cycle. Finally, cationic intermediate **C** lost an acetyl cation affording the desired product.

Imidazoheterocycles were privileged skeletons in commercially available drugs, such as alpidem, olprinone, necopidem, which were developed by the modification of imidazoheterocyclic skeletons.

**Scheme 38:**
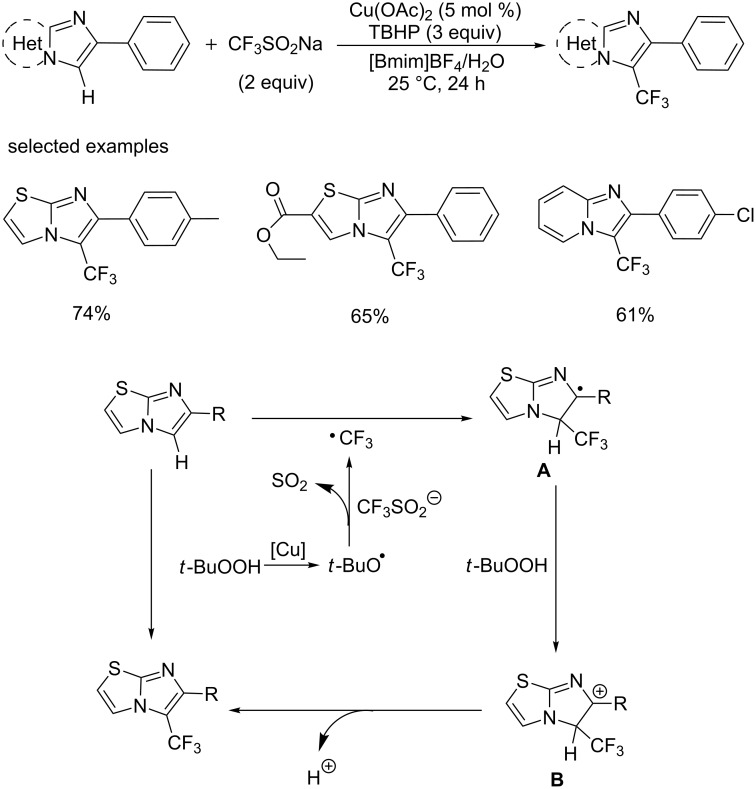
Trifluoromethylation of imidazoheterocycles using CF_3_SO_2_Na as a trifluoromethyl source and the proposed mechanism.

In 2015, the group of Tang [59] developed a regioselective C–H trifluoromethylation of imidazoheterocycles with Langlois' reagent at room temperature (Scheme 38). In order to figure out the insolubility problem of imidazoheterocycles and meet the guiding principles of green chemistry, this reaction was conducted in a recyclable mixed medium of 1-butyl-3-methylimidazolium tetrafluoroborate ([Bmim]BF_4_) and water.

In the presence of catalytic amounts of cupric acetate and TBHP, various substrates, such as 6-arylimidazo[2,1-*b*]thiazoles and imidazopyridines, were compatible with this reaction conditions affording the corresponding analogues in moderate to good yields.

The proposed reaction mechanism is shown in Scheme 38. Initially, the trifluoromethyl radical is generated in situ by the reaction of TBHP with CF_3_SO_2_Na in the presence of copper reagent. Then, the trifluoromethyl radical reacts with substrates affording intermediate **A**, which may be oxidized to a carbocation **B**, followed by an oxidative dehydrogenation process delivering the target product.

#### Copper-mediated/catalyzed direct trifluoromethylation of C(sp)–H

**Scheme 39:**
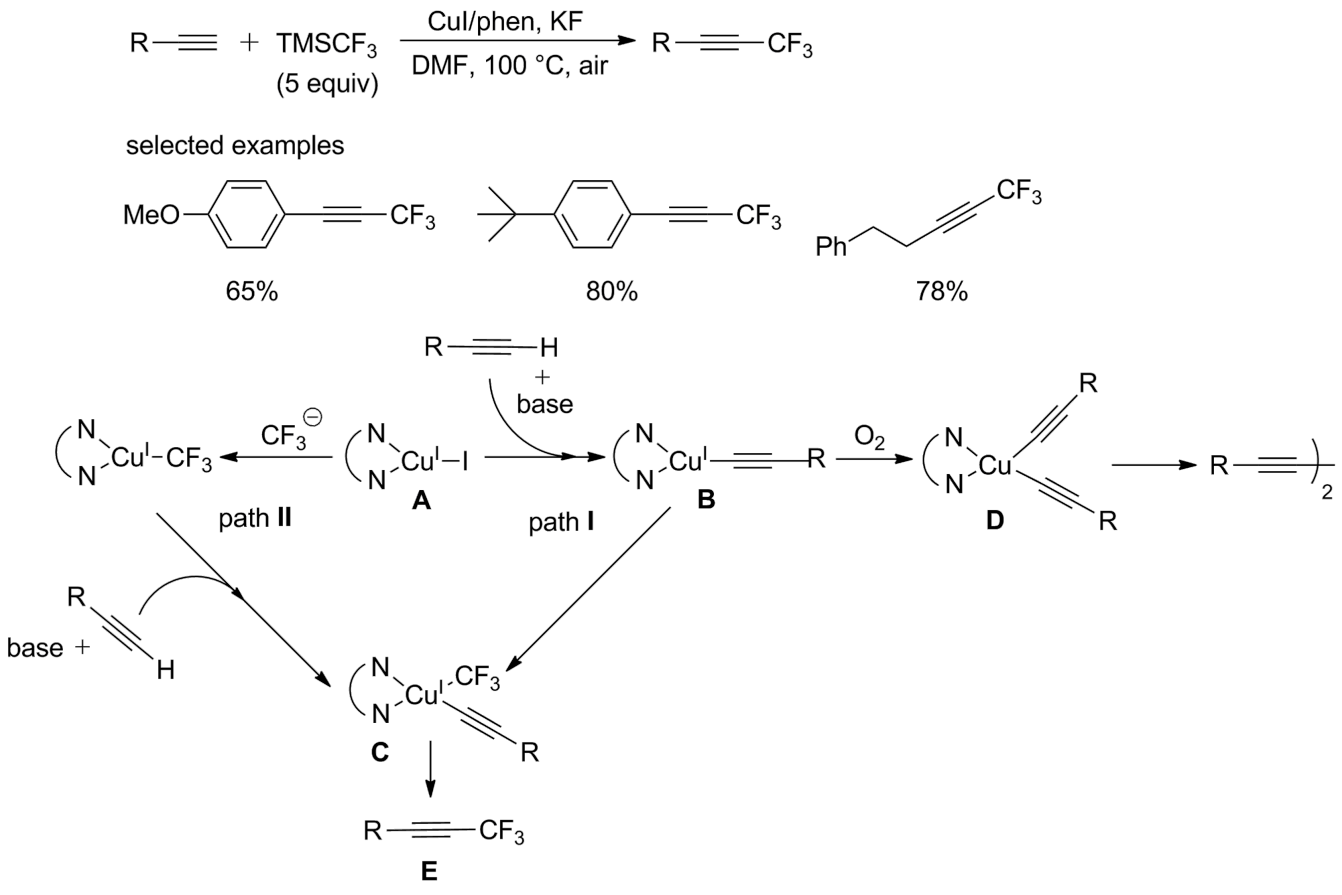
Copper-mediated trifluoromethylation of terminal alkynes using TMSCF_3_ as a trifluoromethyl source and the proposed mechanism.

Trifluoromethylated acetylenes were widely used in medicinal, agrochemical, and material science. In 2010, Qing and coworkers [60] firstly reported a copper-mediated trifluoromethylation of terminal alkynes using TMSCF_3_ as a trifluoromethyl source. (Scheme 39). At the beginning of the experiment, undesired diyne byproduct was formed as major product instead of **E**. The author attributed this phenomenon to the competitive formation of bis-alkynyl–Cu complex **D**, which would produce the undesired diyne byproduct (Scheme 39). In order to solve this problem, the substrates were added by using a syringe pump over a period of 4 h to pregenerated CuCF_3_. Furthermore, phen was introduced to this system to improve the yield. An excess of TMSCF_3_ was required to get a high yield due to the decomposition of TMSCF_3_ under this reaction condition. Aromatic alkynes as well as aliphatic alkynes worked well to give the corresponding products in moderate to good yields. This reaction can tolerate a variety of functionalities, such as alkoxy, amino, ester, and nitro groups.

**Scheme 40:**
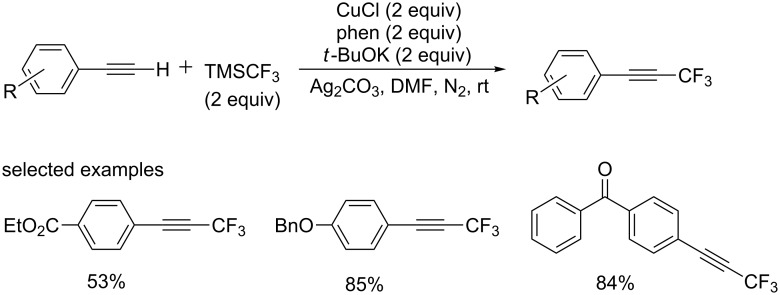
Improved copper-mediated trifluoromethylation of terminal alkynes reported by the group of Qing.

However, a reaction temperature up to 100 °C and the requirement of excessive TMSCF_3_ (bp 55 °C) rendered the former reaction less than ideal. In 2012, the same group [61] developed an improved procedure for the efficient copper-mediated trifluoromethylation of terminal alkynes (Scheme 40). This reaction was conducted at room temperature with a smaller amount of TMSCF_3_.

However, the above-mentioned reaction still required stoichiometric amounts of copper. It is desirable to find novel methods to reduce the Cu loadings to catalytic quantities.

**Scheme 41:**
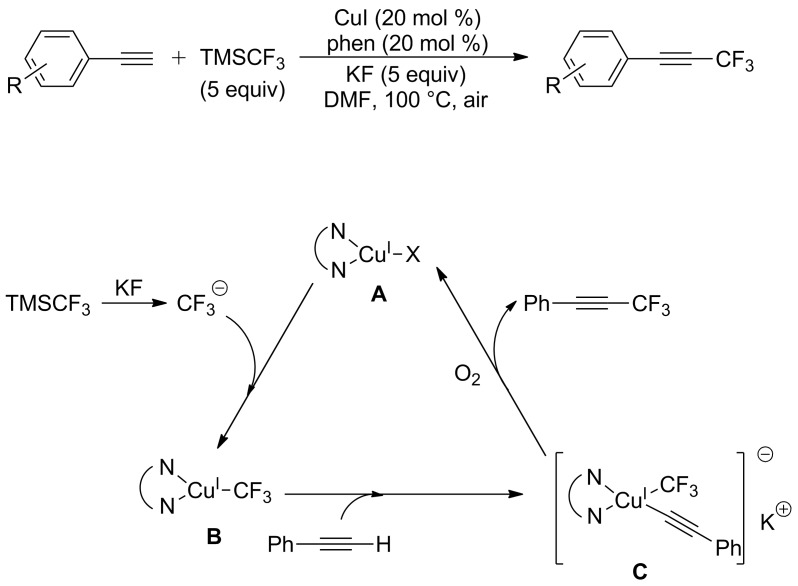
Copper-catalyzed trifluoromethylation of terminal alkynes reported by the group of Qing.

The preliminary mechanistic studies on the above-mentioned work indicated that CuCF_3_ was generated in that transformation. It was found that the generation rate of trifluoromethyl anion was much higher than the next step affording B and CuI. Thus, there is no sufficient recirculated CuI to react with trifluoromethyl before its decomposition. And stoichiometric amounts of copper were required in this conversion. To obviate the problem, Qing and co-workers [62] adopted a new addition method. Adding a portion of TMSCF_3_ (2 equiv) to the mixture afforded CuCF_3_. And both terminal alkynes and the rest of TMSCF_3_ were added to the reaction mixture slowly using a syringe pump. Various arylalkynes bearing electron-donating or -withdrawing groups were converted to the desirable products in good to high yields (Scheme 41).

**Scheme 42:**
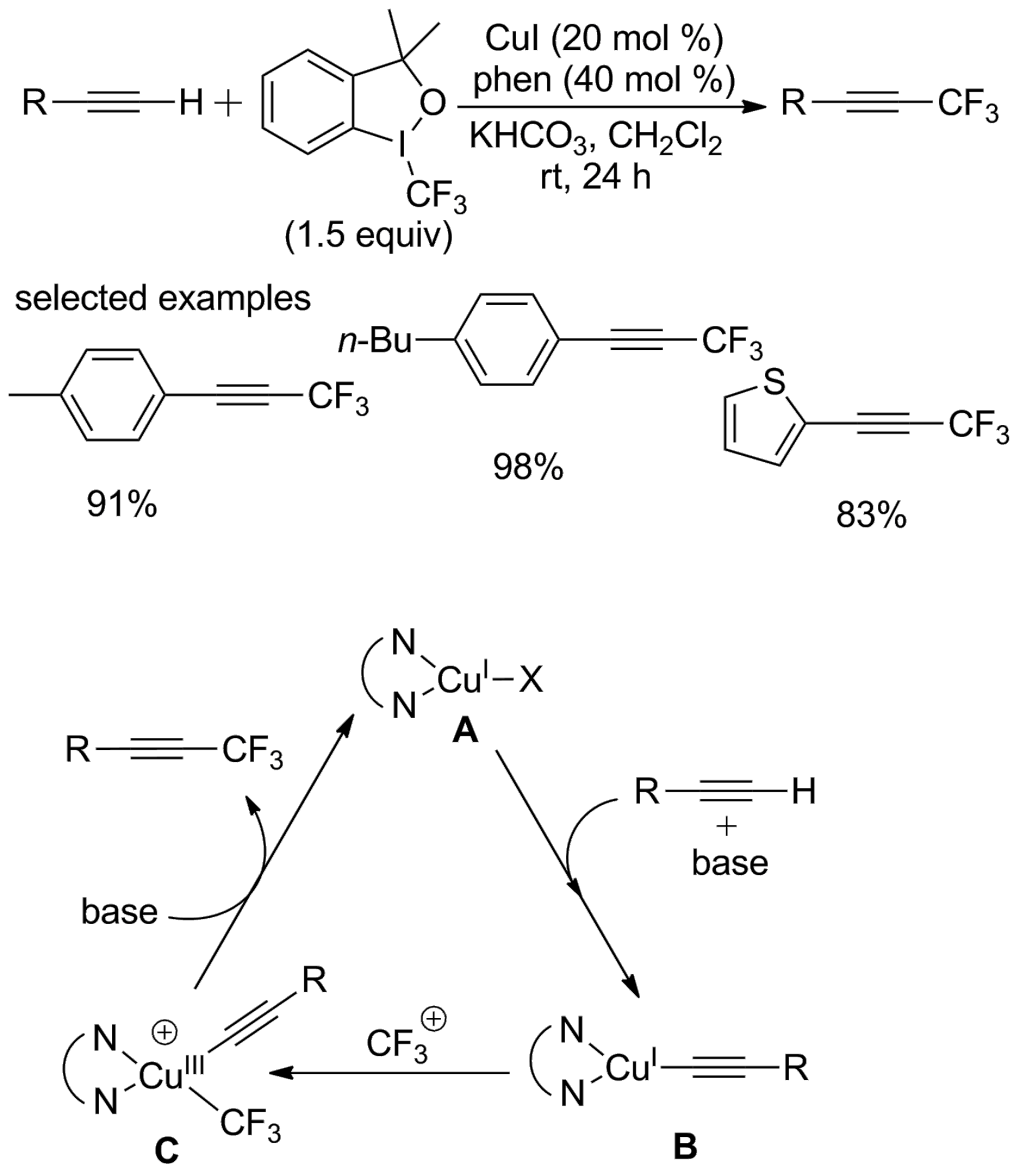
Copper-catalyzed trifluoromethylation of terminal alkynes using Togni’s reagent and the proposed mechanism.

In 2012, the group of Weng and Huang [63] achieved a trifluoromethylation of terminal alkynes using Togni’s reagent (Scheme 42). This reaction was conducted at room temperature with catalytic amounts of copper salt. The high reactivity was observed with phenylacetylenes bearing electron-donating groups. This reaction can tolerate a variety of functionalities, such as alkoxy, amino and halide groups.

The author proposed a plausible mechanism depicted in Scheme 42. At first, a dinitrogen ligated complex (*N,N*)CuX **A** is generated in situ by the reaction of CuI with phen. Then, a copper(I)-acetylide species **B** is formed through the coordination/deprotonation of the alkyne in the presence of base, followed by oxidative addition of CF_3_^+^ and reductive elimination providing the desired product. The copper complex **A** was regenerated to complete the catalytic cycle.

**Scheme 43:**
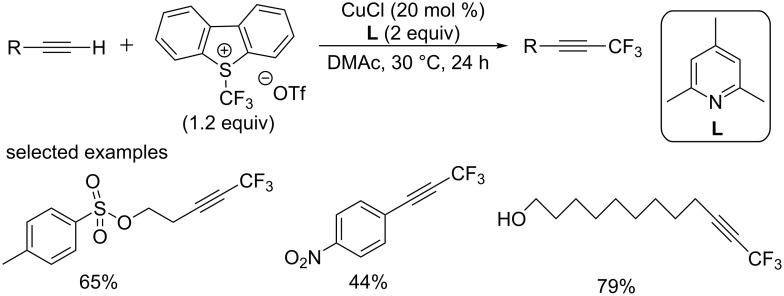
Copper-catalyzed trifluoromethylation of terminal alkynes using Umemoto’s reagent reported by the group of Fu and Guo.

In the same year, the group of Fu and Guo [64] described a trifluoromethylation reaction of terminal alkynes using Umemoto’s reagent as a trifluoromethyl source (Scheme 43). Various terminal alkynes underwent smoothly to provide the corresponding products in moderate to good yields at room temperature. Many synthetically important functional groups were tolerated in these conditions, such as sulfonate, nitro, ester, amide, ether, and even unprotected hydroxy groups. In addition, arene rings bearing chloro and iodo groups did not interfere with the transformation, which promised further conversion at the halogenated positions. It was notable that substrates bearing sulfonate group had a high reactivity.

**Scheme 44:**
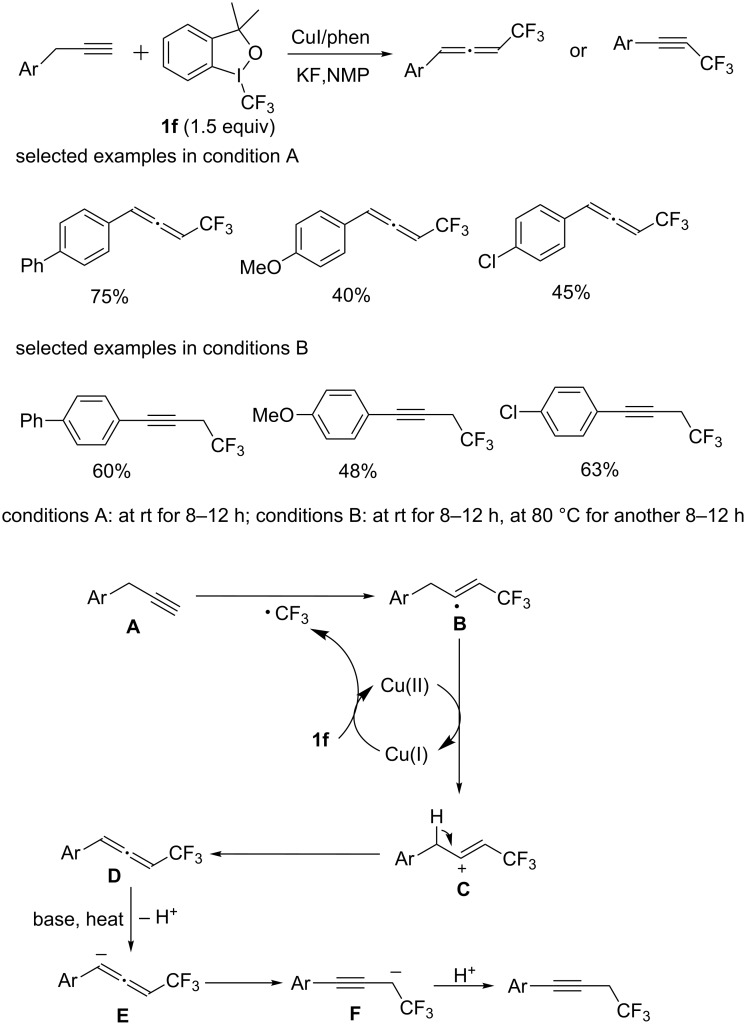
Copper-catalyzed trifluoromethylation of 3-arylprop-1-ynes reported by Xiao and Lin and the proposed mechanism.

Recently, the group of Xiao and Lin [65] developed a copper-catalyzed C–H trifluoromethylation of 3-arylprop-1-ynes to provide (trifluoromethyl)allenes and propargyl trifluoromethanes (Scheme 44). This reaction was the first reported example for selective construction of allenic C(sp^2^)−CF_3_ and propargyl C(sp^3^)−CF_3_ bonds by modifying the reaction conditions. The ratio of propargyl trifluoromethanes increased dramatically when a solution of allenic products under conditions A was further heated. Compared with previous reactions of construction of allenic C(sp^2^)−CF_3_ and propargyl C(sp^3^)−CF_3_ bonds, this method combined selectivity and efficiency and showed atom economy by avoiding the requirement of the prefunctionalization of the substrates.

Regarding the reaction mechanism, the authors postulated the following plausible reaction pathways (Scheme 44). Initially, the CF_3_ radical is generated by the reaction of Togni’s reagent with Cu(I) salt, which is trapped by 3-arylprop-1-yne to produce the radical intermediate **B**. Then, oxidation occurs to intermediate **B** by Cu(II) providing the cationic intermediate **C** and CuI, followed by the deprotonation of a benzyl proton in intermediate **C** giving the allenic product **D**. At high temperature, deprotonation of allene **D** prefers to form anion **E** in the presence of KF, which is converted to anion **F** through a resonance effect. Protonation of intermediate **F** would then furnish the propargyl trifluoromethanes.

## Conclusion

In the past few years, the field of copper-mediated trifluoromethylation of aromatic and aliphatic compounds including heterocycles have experienced significant advances. In parallel with this field, the trifluoromethylation catalyzed by other transition metals like Pd, Ag, Fe and photocatalysts, metal-free methods also have made tremendous advance. For trifluoromethylation of prefunctionalized substrates, these developments have expanded the substrate scope and provided milder conditions, but this field still suffered from limited and expensive trifluoromethylation reagents. Moreover, despite great progress have been made, current methods still lack enough efficiency for their general use in practical large-scale manufacturing.

Likewise, recently the direct trifluoromethylation of aliphatic and aromatic hydrocarbons as well as heterocycles are just starting out and remain a challenge. These methods have often the drawback of generating mixtures of regioisomers. Future efforts will be focus on developing efficient and less expensive reagents, along with a better understanding of mechanisms, improving the regioselectivity and enantioselectivity in these trifluoromethylation processes.
